# Dynamics and Forecasting of Emergency Department Presentations: An Integrative Approach Based on Statistical Analysis and Time-Series Models

**DOI:** 10.3390/healthcare14172800

**Published:** 2026-09-01

**Authors:** Lorena Mihaela Sas, Ana-Maria Camelia Popescu, Vlad Ionuț Ducu, Liviu Popescu, Ion Buligiu, Laurențiu-Stelian Mihai, Mihai Dragoș Cojocaru, Teodor Nicușor Sas

**Affiliations:** 1 Faculty of Medicine, University of Medicine and Pharmacy of Craiova, Str. Petru Rares 2, 200349 Craiova, Dolj, Romania; lori_mika@yahoo.com (L.M.S.); popescuanamaria78@yahoo.com (A.-M.C.P.); vladionutducu@gmail.com (V.I.D.); teodorsas@yahoo.com (T.N.S.); 2 Faculty of Letters, University of Craiova, Str. A.I. Cuza nr 13, 200585 Craiova, Dolj, Romania; 3 Faculty of Economics and Business Administration, University of Craiova, Str. A.I. Cuza nr 13, 200585 Craiova, Dolj, Romania; liviu.popescu@edu.ucv.ro (L.P.); ion.buligiu@edu.ucv.ro (I.B.); 4 Independent Researcher, 8401 Winterthur, Switzerland; dragoscjcr@gmail.com

**Keywords:** health management, ED presentation, forecasting, time-series analysis

## Abstract

**Background/Objectives**: Emergency Department (ED) attendance is characterized by substantial temporal variability, creating challenges for workforce scheduling, resource allocation and short-term operational planning. This study aimed to characterize the temporal patterns of daily ED presentations and to evaluate the short-term forecasting performance of SARIMA and Prophet models in a Romanian hospital setting. **Methods**: Daily ED visits recorded at the Craiova County Emergency Clinical Hospital between January 2025 and April 2026 were analyzed (485 consecutive days). The analytical framework combined descriptive statistics, normality assessment, the Kruskal–Wallis test, and post hoc Mann–Whitney U tests with Holm correction and time-series modeling. A SARIMA(1,0,5)(0,0,1)_7_ model incorporating a Romanian legal-holiday dummy and weekly seasonality and a Prophet model incorporating Romanian holidays and weekly seasonality without yearly seasonality were evaluated using a common rolling-origin out-of-sample framework. Predictive accuracy was assessed using RMSE and MAPE, while differences in forecasting performance were examined using the Diebold–Mariano test with a HAC/Newey–West correction. **Results**: ED attendance differed significantly across weekdays (H(6) = 52.365, *p* < 0.001), with the highest mean attendance observed on Mondays (257.6 patients/day). Post hoc analysis confirmed significant differences between several weekday distributions after Holm adjustment, supporting a structured weekly pattern. Time-series analysis demonstrated serial dependence and recurrent weekly seasonality. Both forecasting models showed good short-term predictive performance across the four common validation windows. Over the complete out-of-sample period of 1–30 April 2026, SARIMA achieved an RMSE of 22.59 patients and a MAPE of 7.46%, compared with 23.54 patients and 7.56%, respectively, for Prophet. Although the aggregate results slightly favored SARIMA, the difference in predictive accuracy was not statistically significant (Diebold–Mariano test, *p*-value = 0.575). **Conclusions**: Daily ED attendance at SCJU Craiova exhibits systematic weekly variation, serial dependence and calendar-related effects that can be incorporated into short-term forecasting models. SARIMA and Prophet provided comparable out-of-sample predictive accuracy, with neither model demonstrating statistically significant superiority. These findings support the use of short-term forecasting as an operational planning tool for anticipating variations in ED demand and informing adaptive resource allocation.

## 1. Introduction

Emergency services represent one of the most dynamic components of healthcare systems, characterized by substantial temporal variability in patient presentations, continuous operational demands and the need for adaptive allocation of medical resources. Over the past two decades, emergency department (ED) overcrowding has emerged as a major public health and healthcare management challenge and has been associated with prolonged waiting times, reduced quality of care, staff burnout, and an increased risk of adverse outcomes [1,2]. Importantly, ED overcrowding is a multidimensional phenomenon resulting from the interaction between patient demand and healthcare system capacity, including staffing levels, bed availability, length of stay, treatment delays and patient-flow processes. Therefore, the volume and temporal distribution of ED presentations represent important components of emergency care demand but should not be considered synonymous with ED overcrowding.

Understanding the temporal dynamics of ED attendance is an important prerequisite for effective operational planning. Emergency departments must accommodate substantial fluctuations in patient demand while maintaining adequate levels of staffing, equipment and other healthcare resources. Previous studies conducted in European and North American healthcare systems have demonstrated recurrent temporal patterns in ED attendance, including weekly and seasonal variations, as well as associations with calendar effects, accessibility of healthcare services, socioeconomic characteristics and population healthcare-seeking behavior [1,2]. However, although such factors may contribute to variations in emergency care utilization, their specific effects cannot be established from attendance data alone.

In the Romanian healthcare system, emergency departments represent an important point of access to hospital-based medical care. Previous evidence has reported a substantial proportion of ED presentations classified as lower-acuity cases, including patients assigned to the green triage code, and has suggested that a proportion of emergency presentations may potentially be managed in outpatient or primary-care settings. Nevertheless, such observations should be interpreted in the context of the broader organization of healthcare services and should not be regarded as direct evidence of ED overcrowding or as proof that primary-care accessibility is the sole determinant of emergency service utilization. The present study therefore focuses specifically on the statistical and temporal characteristics of daily ED attendance at the emergency department of the Craiova County Emergency Clinical Hospital (SCJU Craiova).

Accurate forecasting of healthcare demand requires a comprehensive understanding of the temporal structure of emergency care utilization. The international literature on ED demand prediction has developed along three broadly distinguishable methodological lines. 

The first and most established line applies classical statistical time-series methods. Autoregressive models such as ARIMA and seasonal ARIMA (SARIMA) are widely used for forecasting emergency department demand because of their ability to capture serial dependence and seasonal patterns in healthcare time series [3,4]. Jones et al. [3] demonstrated stable weekly attendance patterns and reliable short-term forecasts of daily patient volume in United States emergency departments, while Abdel-Aal and Mangoud [4] showed that seasonal ARIMA specifications adequately represent seasonality and serial dependence in healthcare utilization series. The systematic review by Wargon et al. [5] confirmed that regression- and ARIMA-type models constitute the predominant approaches in this field and that calendar variables, particularly day-of-week indicators, account for a substantial share of the explained variability in daily attendance; the subsequent and more extensive review by Gul and Celik [6], covering 102 studies, reached comparable conclusions regarding the continued prevalence of time-series methods in ED forecasting. Later work has extended these specifications with exogenous information: Whitt and Zhang [7] reported that a SARIMAX model combining internal temporal dependence with calendar and meteorological regressors outperformed both simpler benchmarks and a neural-network alternative, and Sudarshan et al. [8] reached similar conclusions when evaluating calendar and weather information across several competing forecasting methods.

A second line of research applies decomposition-based and machine-learning approaches. Prophet, which represents a series as an additive combination of trend, seasonal and holiday components [9], has been applied to hospital bed occupancy [10] and to hospital infection surveillance data [11], with predictive performance that is competitive with, but not systematically superior to, classical seasonal models. Comparative evaluations of machine-learning methods applied to emergency care demand have similarly indicated that flexible algorithms do not consistently outperform well-specified statistical benchmarks, particularly when the available series are of moderate length [12]. Rostami-Tabar and Ziel [13] further showed that public holidays and other special events constitute a distinct source of forecast error in emergency department series, and that the manner in which such calendar effects are represented has a material influence on predictive accuracy.

A third and more recent line of enquiry concerns the evaluation framework itself rather than the models. Comparisons based solely on aggregate error measures such as RMSE and MAPE cannot establish whether an observed difference in accuracy is statistically distinguishable from sampling variation. Formal procedures for comparing predictive accuracy, in particular the Diebold–Mariano test [14], applied together with rolling-origin evaluation over identical forecast windows and under identical information sets, are required before claims of model superiority can be supported. Such procedures nevertheless remain comparatively infrequent in the applied emergency-care forecasting literature, in which conclusions are often drawn from point estimates of forecast error alone [6].

Three gaps emerge from this body of work. First, comparatively little attention has been given to combining the distributional analysis of weekday attendance with formal nonparametric testing and multiple-comparison control within a single study. This is particularly relevant in healthcare settings in which daily attendance distributions may depart from normality and in which differences between weekdays may not be adequately characterized by comparisons of means alone. Second, direct comparisons between competing forecasting models are frequently conducted under non-equivalent conditions—differing training periods, forecast horizons or calendar information—with the consequence that observed differences may reflect the evaluation design rather than genuine differences in model performance; such comparisons are moreover rarely accompanied by a formal test of equal predictive accuracy. Third, the empirical evidence base is geographically uneven. The majority of published ED forecasting studies originate from Western European, North American and Asian healthcare systems, whereas evidence from Eastern European and specifically Romanian settings—which differ in the organization of primary care and in patterns of access to hospital-based emergency services—remains comparatively limited.

The present study addresses this gap through an integrative analysis of daily patient presentations at the ED of SCJU Craiova during the period 1 January 2025–30 April 2026. The analytical framework combines descriptive statistics, distributional assessment, nonparametric hypothesis testing and time-series forecasting. Differences in ED attendance across the seven weekdays are assessed using the Kruskal–Wallis test. Following a statistically significant global result, pairwise Mann–Whitney U tests with Holm correction are used to identify specific weekday differences while controlling the family-wise error rate arising from multiple comparisons. The temporal properties of the attendance series are further examined with respect to serial dependence and weekly seasonality. 

For forecasting, the study employs a SARIMA(1,0,5)(0,0,1)_7_ model and a Prophet model. The SARIMA specification incorporates Romanian legal-holiday effects through an explicit dummy variable, while Prophet incorporates the Romanian holiday calendar, together with weekly seasonality but no yearly seasonality. Thus, both approaches account for calendar-related effects, although through different modeling structures. This provides a more balanced comparison of the two forecasting approaches.

The predictive performance of the two models is evaluated using a common rolling-origin, out-of-sample framework. Both models are estimated using only information available up to each respective forecast origin and are evaluated over the same four successive forecast windows covering 1–30 April 2026. Forecast accuracy is assessed using the Root Mean Square Error (RMSE) and Mean Absolute Percentage Error (MAPE). In addition to window-specific performance measures, aggregate RMSE and MAPE are calculated over the complete April evaluation period. To determine whether observed differences in predictive accuracy represent statistically significant differences between the models, the Diebold–Mariano test is applied using squared-error loss and a HAC/Newey–West variance correction.

Addressing these gaps, the present study makes three contributions. First, it integrates the distributional and inferential characterization of daily ED attendance—descriptive analysis, normality assessment, the Kruskal–Wallis test and post hoc Mann–Whitney U tests with Holm correction—with short-term time-series forecasting within a single analytical framework, thereby linking the identification of recurrent demand patterns to their predictive assessment. Second, it compares a classical seasonal statistical model with a decomposition-based forecasting approach under strictly equivalent conditions: identical forecast origins, identical horizons, identical observed outcomes and equivalent calendar information, with the resulting difference in accuracy assessed by means of the Diebold–Mariano test with a HAC/Newey–West correction and a sensitivity analysis across alternative lag specifications. Third, it provides evidence from a Romanian county emergency hospital, thereby extending the geographical coverage of the ED forecasting literature to an Eastern European setting for which comparable published analyses are scarce.

The primary objective of this study is to investigate the temporal dynamics and statistical characteristics of daily patient presentations at the ED of SCJU Craiova and to evaluate short-term forecasting models capable of supporting evidence-based operational planning.

The specific objectives are as follows:

- To characterize the statistical distribution and temporal variability of daily ED attendance using descriptive and inferential statistical methods;
- To assess whether significant differences exist between weekdays in terms of patient attendance volume and distributional characteristics;
- To identify which weekday pairs differ significantly following a statistically significant Kruskal–Wallis test using post hoc Mann–Whitney U tests with Holm correction for multiple comparisons;
- To examine the temporal properties of the attendance series, including weekly seasonality, autocorrelation and serial dependence;
- To develop and evaluate forecasting models based on SARIMA and Prophet methodologies for predicting daily ED attendance;
- To compare the predictive performance of SARIMA and Prophet using a common rolling-origin, out-of-sample evaluation framework with equivalent calendar information;
- To assess whether the observed differences in predictive accuracy between the two models are statistically significant using the Diebold–Mariano test with HAC/Newey–West correction;
- To derive evidence-based implications for resource planning, workforce scheduling and adaptive management in response to fluctuations in ED demand.

Based on these objectives, the following research hypotheses were developed:

**H1:**  *There are statistically significant differences between weekdays in the distribution of patient visits to the SCJU Craiova ED*.

**H2:**  *At least one pair of weekdays differs significantly in ED attendance distribution following post hoc pairwise comparisons with Holm correction*.

**H3:**  *The time series of daily ED visits demonstrates significant weekly seasonality and serial dependence*.

**H4:**  *Time-series forecasting models can provide accurate out-of-sample predictions of daily patient attendance at the ED*.

**H5:**  *SARIMA and Prophet exhibit no statistically significant differences in predictive accuracy when forecasting short-term emergency department attendance*.

## 2. Literature Review

Emergency department (ED) overcrowding and variations in ED patient attendance represent distinct but interrelated challenges in contemporary healthcare management. While ED overcrowding refers primarily to a mismatch between patient demand and the available capacity and resources, variations in ED attendance concern the temporal distribution and volume of patient presentations. From both epidemiological and operational perspectives, the international literature has extensively investigated these phenomena. Over recent decades, numerous studies have demonstrated that ED attendance follows recurrent temporal patterns influenced by seasonal factors, accessibility of primary healthcare services, the sociodemographic structure of the population and regional epidemiological dynamics. Patient presentations are therefore not randomly distributed but may exhibit serial dependence and stable seasonal effects, providing a rationale for the use of statistical and predictive methods to characterize demand patterns and support healthcare resource planning.

One of the most influential studies on forecasting emergency department demand was conducted by Jones et al. [3], who analyzed daily patient flows using autoregressive and time-series regression models applied to EDs in the United States. Their findings proved the existence of stable weekly attendance patterns and showed that SARIMA models can provide robust short-term forecasts of patient volume with relatively low prediction errors and direct applicability to operational management. Their study identified a significant increase in patient attendance on Mondays and in proximity to weekends, a phenomenon frequently referred to in the literature as the “Monday effect” or “post-weekend backlog effect” [3].

A major contribution to the field of emergency department time-series modeling was made by Abdel-Aal and Mangoud [4], who employed ARIMA models to forecast monthly ED demand at a primary health care clinic. Their research demonstrated that SARIMA models effectively capture seasonality and serial dependence in epidemiological time series and provide reliable estimates of operational workload. The study emphasized the importance of seasonal components and showed that incorporating autoregressive and moving-average terms significantly improves predictive performance [3].

An important branch of the recent literature studies the phenomenon of ED overcrowding and its consequences for healthcare quality. Morley et al. [2] conducted a comprehensive systematic review investigating the causes and consequences of ED overcrowding. Their findings demonstrated that overcrowding is associated with increased mortality, treatment delays, prolonged waiting times, and reduced patient satisfaction. The authors identified limited access to primary care, delayed hospital admissions, and temporal fluctuations in patient demand as major contributing factors [2].

The impact of ED overcrowding on hospitalization duration and treatment delays has also been investigated by McCarthy et al. [15], who demonstrated that crowding leads to significant delays in treatment even among patients with severe medical conditions. Their findings suggested that overcrowding generates cumulative effects that influence the entire clinical and logistical workflow of the hospital [15].

Whitt and Zhang [7] developed and compared forecasting methods for predicting both daily patient arrivals and real-time hourly occupancy levels in an emergency department using publicly available data from the Rambam Health Care Campus in Haifa, Israel, collected between 2004 and 2007. Their study extended a previous stochastic model by utilizing a substantially longer dataset (200 weeks instead of 25), allowing for the identification of long-term growth trends in both arrival processes and length-of-stay distributions, as well as dependence between consecutive daily arrival totals. Five one-day-ahead forecasting approaches were evaluated: a dynamic moving-average model, a SARIMA model, a regression model incorporating calendar and meteorological variables, a SARIMAX model, and a multilayer perceptron (MLP) neural network. The results showed that the day-of-week effect explained the majority of variation in demand, while holiday indicators and daily maximum and minimum temperatures contributed significantly to forecasting accuracy. The SARIMAX model, which combined internal time-series dependence with exogenous variables, achieved the best performance, yielding a test-set mean squared error of 191.8 and a mean absolute percentage error (MAPE) of 8.4%, representing an improvement of approximately 25%. Notably, the neural network did not outperform the well-structured time-series models, a result attributed by the authors to the relatively limited size of the dataset.

In recent years, advances in machine learning and artificial intelligence have led to the development of new predictive models for epidemiological and operational time series. One widely adopted model is Prophet, developed by Meta Platforms, which employs an additive decompositional framework to capture trends and complex seasonal patterns. Taylor and Letham [9] demonstrated that Prophet provides strong predictive performance for time series characterized by seasonality and nonlinear variations.

The study of Fattouh et al. [10] investigates the use of the Prophet model for mid-term hospital bed occupancy forecasting, utilizing daily data collected from the University Medical Center Freiburg between 2010 and 2023. The model incorporated external factors such as public holidays and the impact of the COVID-19 pandemic and was evaluated across forecasting horizons ranging from 30 to 180 days. The results demonstrated high predictive accuracy, with Mean Absolute Percentage Error (MAPE) values ranging from 3.21% to 3.53%, achieving performance comparable to or even exceeding that of considerably more complex machine learning models. Moreover, Prophet offers the advantage of interpretability by clearly identifying the effects of long-term trends, seasonality, and holiday-related variations on hospital bed occupancy. The study concludes that simple and transparent models such as Prophet can provide highly accurate forecasts of hospital bed occupancy while offering lower implementation and maintenance costs, faster deployment, and greater operational value than more complex neural network-based approaches.

Also, the study of Chen & Zhou [11] compared four time-series forecasting models—SARIMA, ETS, Prophet, and NNETAR—for predicting infection rates caused by multidrug-resistant organisms (MDROs) in a tertiary hospital in China. The analysis was based on monthly data collected between 2014 and 2023, with forecasts generated for the year 2024. The results indicated that the SARIMA model provided the most robust and reliable forecasts, demonstrating strong predictive accuracy and satisfactory residual diagnostics. Although the ETS model achieved some of the lowest forecasting errors, diagnostic tests raised concerns regarding its stability and robustness. The Prophet and NNETAR models were able to capture certain characteristics of the data but showed lower overall performance compared with SARIMA. The study concludes that SARIMA is the most suitable model for short- to medium-term forecasting of MDRO infection trends. The other models, while less reliable as standalone forecasting tools, may still serve as complementary approaches for trend validation and sensitivity analyses to support hospital infection control planning and decision-making.

Recent literature increasingly emphasizes the importance of integrating predictive analytics into emergency healthcare management. Wargon et al. [5] demonstrated that predictive methods can reduce waiting times and optimize the allocation of human and logistical resources in emergency services. Likewise, studies by Sun et al. [1] showed that predictive models applied at the triage stage can substantially improve the estimation of hospital admission requirements and the management of patient flows.

Taken together, the studies reviewed above differ substantially in the outcome forecast (daily ED arrivals, monthly clinic attendance, hospital bed occupancy, infection incidence), in the level of temporal aggregation, in the forecast horizon, in the exogenous information incorporated and in the accuracy measures reported. This heterogeneity limits the extent to which their numerical results can be placed on a common scale and compared directly. What can meaningfully be compared, and what is of direct relevance to the present study, concerns the design of the analyses rather than the magnitude of the errors they report. In this respect, three features recur across the literature. Distributional characterization of attendance and formal forecasting are typically undertaken separately rather than within a single analytical framework. Competing models are frequently evaluated under non-equivalent training periods, forecast horizons or information sets, and the resulting differences in accuracy are rarely subjected to a formal test of equal predictive accuracy. Finally, the geographical coverage of the evidence base remains concentrated in Western European, North American and Asian healthcare systems. These three observations define the gap addressed by the present study and are stated explicitly in Section 1.

## 3. Research Methodology

**Figure 1:**
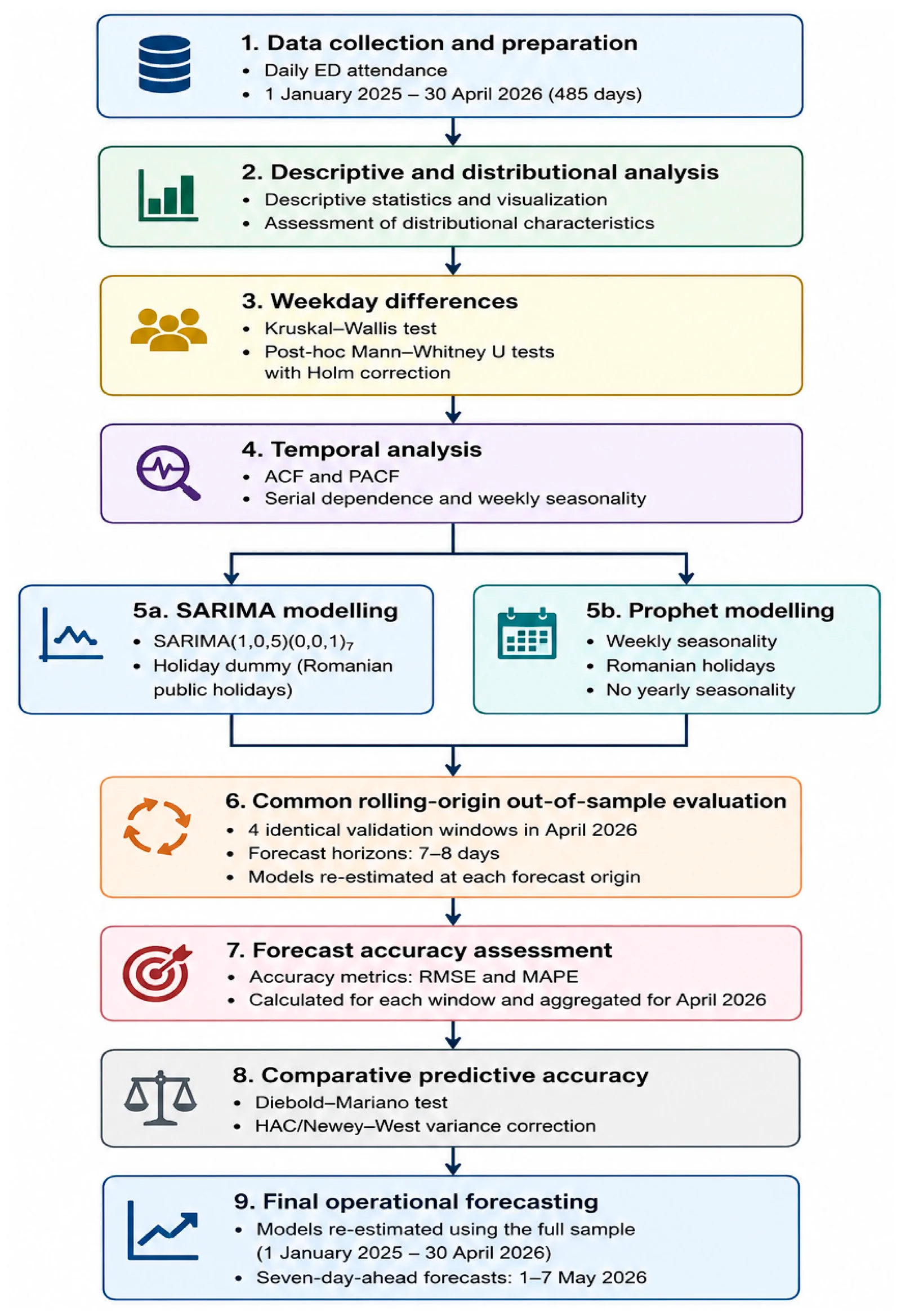
Analytical workflow.

The methodological workflow followed a sequential analytical framework, beginning with data preparation and descriptive analysis and proceeding through distributional comparisons, temporal analysis, forecasting model development, common out-of-sample validation and comparative assessment of predictive accuracy. The overall analytical workflow is summarized in Figure 1.

### 3.1. Study Design and Data

The study utilized a retrospective database derived from the administrative records of the emergency department (ED) of the Craiova County Emergency Clinical Hospital (SCJU Craiova). The analyzed data covered the period from 1 January 2025 to 30 April 2026 and included all patient visits registered in the emergency service during the study period.

The statistical unit of analysis was the calendar day and the primary variable of interest was the total number of patients presenting daily to the ED, regardless of triage category, diagnosis, or admission status. Accordingly, each observation in the dataset represented a single calendar day and contained the aggregated number of ED visits recorded on that date.

The study period comprised 485 consecutive days, providing a sufficiently large dataset for assessing temporal variability, identifying recurrent weekly patterns and developing and evaluating short-term forecasting models. For each observation, additional derived variables were generated, including the day of the week and calendar-related indicators used in the statistical and forecasting analyses.

The data underwent a preliminary verification and validation process to identify missing observations and obvious data anomalies. The analytical framework included descriptive and distributional analyses, nonparametric statistical testing, time-series modeling, and out-of-sample forecasting evaluation. The database reflects real-world patterns of emergency department utilization and provides a basis for examining temporal variation in ED attendance and forecasting short-term changes in patient demand.

### 3.2. Distributional Analysis and Normality Assessment

Descriptive statistics were used to characterize the distribution and temporal variability of daily ED attendance. Measures of central tendency and dispersion were calculated for the overall series and for the seven weekday groups. Skewness and kurtosis coefficients were also examined to provide additional information on the shape of the attendance distributions.

Prior to the application of inferential statistical procedures, the normality of the data distributions was assessed using the Shapiro–Wilk test [16]. The test evaluates the null hypothesis that the observations are drawn from a normally distributed population. Failure to reject the null hypothesis was interpreted as indicating insufficient evidence against normality at the predefined significance level of α = 0.05.

As a complementary diagnostic, the Jarque–Bera test was also considered for assessing departures from normality based on the skewness and kurtosis of the distribution. The test evaluates the null hypothesis of normality, with a *p*-value below 0.05 indicating evidence against this assumption. The Jarque–Bera test was used as a complementary diagnostic rather than as a substitute for the Shapiro–Wilk test.

### 3.3. Nonparametric Analysis of Weekday Differences

The Mann–Whitney U test [17] is a nonparametric procedure used to compare the distributions of two independent groups. It is particularly appropriate when the assumptions required for parametric procedures are not satisfied. The method is based on the ranks of the pooled observations and evaluates whether observations from one group tend to have systematically higher or lower ranks than those from the other group. The null hypothesis states that the two groups have the same distribution, whereas the alternative hypothesis indicates a difference between their distributions.

The Kruskal–Wallis test [18] was used to assess whether daily ED attendance differed significantly across the seven days of the week. The test is a nonparametric alternative to one-way analysis of variance and evaluates the null hypothesis that the distributions of the outcome variable are identical across the groups. The alternative hypothesis states that at least one group differs from the others. A statistically significant Kruskal–Wallis result indicates the presence of differences among the weekday groups but does not identify the specific pairs responsible for the overall difference.

Following a statistically significant Kruskal–Wallis test, pairwise Mann–Whitney U tests were performed to identify the specific weekday pairs exhibiting statistically significant differences in ED attendance. Because seven weekday groups generate 21 pairwise comparisons, the Holm correction was applied to the resulting *p*-values to control the family-wise error rate associated with multiple testing. The Holm procedure sequentially adjusts the significance threshold according to the ordered *p*-values and provides control of the family-wise error rate while being less conservative than the traditional Bonferroni correction.

Thus, the weekday analysis consisted of an overall Kruskal–Wallis test followed, when statistically significant, by pairwise Mann–Whitney U comparisons with Holm adjustment. This two-stage approach allowed assessment of both the overall presence of weekday differences and the specific weekday pairs contributing to those differences. 

### 3.4. SARIMA Modeling

For the analysis and forecasting of daily ED attendance, the Seasonal Autoregressive Integrated Moving Average (SARIMA) methodology was employed. SARIMA is a widely used statistical approach for modeling time series characterized by serial dependence and recurrent seasonal patterns. 

The general SARIMA model is denoted as SARIMA(*p*,*d*,*q*)(*P*,*D*,*Q*)_s_, where *p*, *d*, and *q* represent the orders of the non-seasonal autoregressive, differencing, and moving-average components, respectively. The parameters *P*, *D*, and *Q* represent the corresponding seasonal components, while *s* denotes the seasonal period. In the present study, the seasonal period was set to seven observations to represent the weekly pattern in daily ED attendance.

The modeling process included assessment of stationarity using the Augmented Dickey–Fuller (ADF) test [19], followed by examination of the autocorrelation function (ACF) and the partial autocorrelation function (PACF) to identify the temporal dependence structure and inform candidate model specifications. Candidate models were evaluated using information criteria, including the Akaike Information Criterion (AIC) and Bayesian Information Criterion (BIC), together with parameter significance and residual diagnostic procedures.

Residual adequacy was assessed using the Breusch–Godfrey test [20,21] for serial correlation and the ARCH test for conditional heteroscedasticity. These diagnostics were used to evaluate whether relevant temporal dependence or conditional variance effects remained in the model residuals. Forecasting performance was assessed using the Root Mean Squared Error (RMSE) and Mean Absolute Percentage Error (MAPE).

To account for calendar-related variations associated with Romanian legal holidays, a holiday dummy variable was incorporated into the forecasting specification. Thus, the SARIMA model simultaneously accounted for serial dependence, weekly seasonality and identifiable calendar effects.

### 3.5. Prophet Forecasting

Prophet is an additive forecasting model designed to represent time series through trends, seasonal components, and effects associated with holidays or other specified events [9]. The approach provides a flexible framework for modeling time series with recurrent seasonal patterns and calendar-related variations.

The model can be expressed as follows:

y(t) = g(t) + s(t) + h(t) + ε(t),(1)

where g(t) represents the trend component, s(t) the seasonal component, h(t) the effects associated with holidays or special events, and ε(t) the error term. The trend is estimated using piecewise linear or logistic growth models, while seasonality is modeled using Fourier series, allowing for the capture of complex periodic patterns. A key advantage of Prophet lies in its flexibility in handling missing data, outliers and structural changes in the time series.

In the present study, Prophet was implemented using Python 3.13.15 and the Prophet library. The model incorporated Romanian legal holidays and weekly seasonality, while yearly seasonality was not included. This specification was designed to capture the recurrent weekly structure identified in the ED attendance series while accounting for calendar-related effects.

The Prophet model was evaluated using the same out-of-sample forecasting framework and forecast periods as the SARIMA model. Forecast accuracy was assessed using RMSE and MAPE, and the model also generated uncertainty intervals associated with the forecasts.

### 3.6. Rolling-Origin Out-of-Sample Evaluation

To ensure a fair and temporally consistent comparison between SARIMA and Prophet, predictive performance was assessed using a common rolling-origin out-of-sample evaluation framework. At each forecast origin, both models were estimated using only the observations available up to that point and were subsequently used to generate forecasts for the same future period.

The forecast origin was then moved forward and the procedure was repeated, resulting in multiple successive out-of-sample evaluation windows. This approach allows model performance to be assessed under changing information sets and reduces the risk of obtaining overly optimistic accuracy estimates that may arise from in-sample evaluation.

Both forecasting approaches were evaluated over the same four successive forecast windows covering 1–30 April 2026. The forecast horizons consisted of seven or eight consecutive days, depending on the predefined forecast windows. For each origin, the observed values were retained exclusively for evaluating the corresponding forecasts and were not available to the models during estimation.

The use of identical forecast origins, forecast horizons, observed outcomes and calendar information ensured that SARIMA and Prophet were compared under equivalent out-of-sample conditions. This common evaluation design provides a direct assessment of their relative performance in short-term ED attendance forecasting.

### 3.7. Forecast Accuracy Measures

Forecast accuracy was assessed using the Root Mean Squared Error (RMSE) and Mean Absolute Percentage Error (MAPE). RMSE measures the magnitude of forecast errors while assigning greater weight to larger deviations. MAPE expresses the average absolute forecast error relative to the corresponding observed values and provides a scale-independent measure of predictive accuracy.

Lower values of both RMSE and MAPE indicate better forecasting performance. The two measures provide complementary information: RMSE is more sensitive to large individual forecasting errors, whereas MAPE facilitates interpretation of forecast errors relative to the observed level of ED attendance.

Accuracy measures were calculated separately for each rolling-origin forecast window. In addition, aggregate RMSE and MAPE were calculated over the entire April 2026 out-of-sample evaluation period to provide an overall assessment of predictive performance across the common evaluation horizon.

### 3.8. Diebold–Mariano Test

The Diebold–Mariano (DM) test [14] is a statistical procedure used to compare the predictive accuracy of two competing forecasting models. It evaluates whether the difference between the forecasting losses generated by the two models is statistically significant. The test is based on a loss function applied to the forecast errors and tests the null hypothesis of equal predictive accuracy between the competing models.

The choice of the loss function depends on the objective of the comparison. Squared-error loss is commonly used when larger forecasting errors are intended to receive greater weight. The resulting loss differential represents the difference in forecasting performance between the two models over the evaluation period.

Standard statistical inference for the DM test may be affected when the loss differential exhibits heteroscedasticity or serial correlation, particularly when forecasts are generated for consecutive observations in a time series. To account for these features, the variance of the loss differential can be estimated using a heteroscedasticity- and autocorrelation-consistent (HAC) estimator, such as the Newey–West estimator. This adjustment provides more robust standard errors and more reliable inference regarding differences in predictive accuracy.

The null hypothesis of the DM test states that the two forecasting models have equal predictive accuracy. A statistically significant result provides evidence of a difference in predictive performance, whereas a non-significant result indicates that the observed difference in forecasting errors is not sufficient to establish statistically different predictive accuracy.

## 4. Results

### 4.1. Daily Visit Dynamics in ED

**Figure 2:**
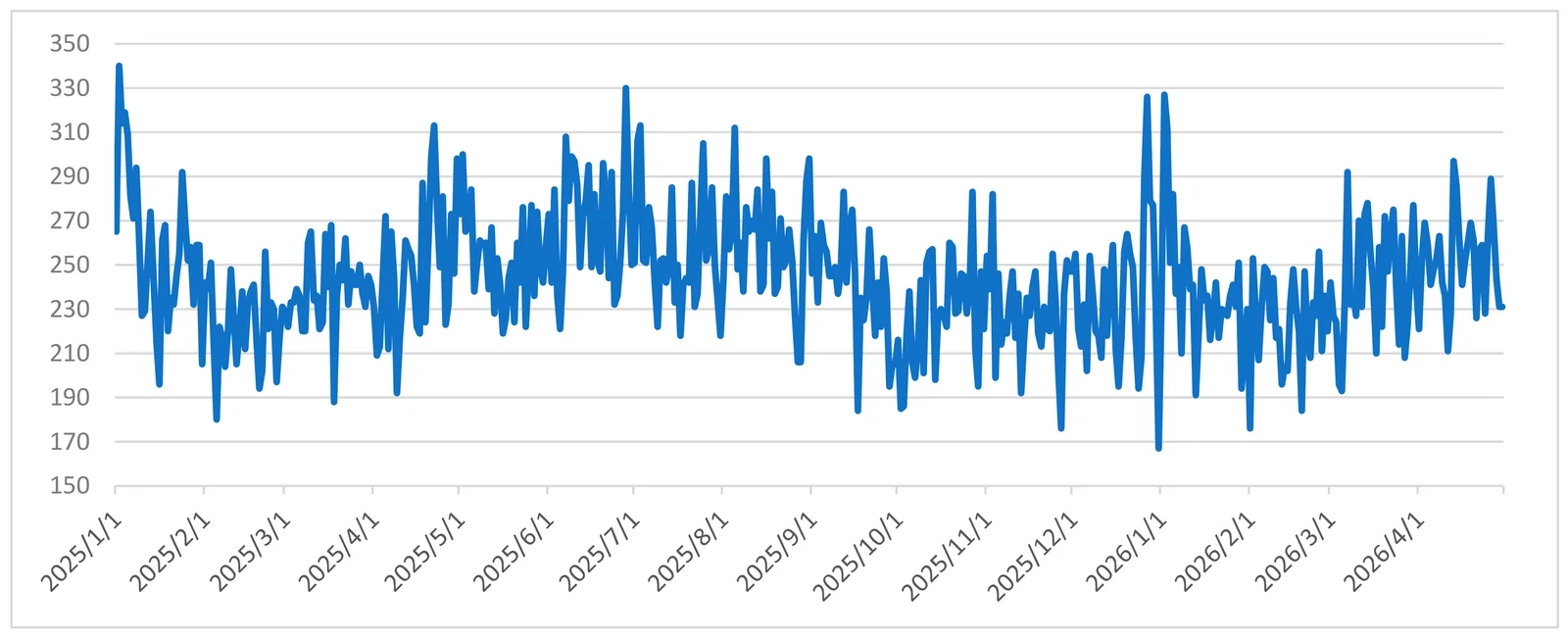
ED visits evolution, authors’ contribution with Microsoft Excel, based on data from Table A5.

The historical data for the period January 2025–April 2026 show substantial variability, with daily ED attendance ranging from a minimum of 167 visits (31 December 2025) to a maximum of 340 visits (2 January 2025) (Figure 2).

The observed variation exhibits a temporal pattern, as summarized in Table 1. The reported values represent descriptive averages calculated from the daily ED visit counts within each specified time interval. The highest average was observed during May–June 2025 (260.7 visits/day), followed by January 2025 (259.1 visits/day) and July–August 2025 (257.3 visits/day), whereas lower average levels were observed during February–March 2025 (231.1 visits/day) and September–November 2025 (232.6 visits/day). These variations provide a descriptive indication of periods with relatively higher or lower ED utilization. However, the potential contributing factors listed in Table 1 should be interpreted as contextual hypotheses rather than as statistically established causal relationships.

This temporal distribution highlights the need for forecasting models capable of accounting for both short-term variability in daily ED visits and recurring seasonal patterns. In this context, forecasting may provide practical support for anticipating staffing and resource requirements.

A potentially relevant source of short-term variation is the Romanian public holiday calendar. In 2025, several holiday periods coincided with increased ED attendance. For example, the government’s decision to establish Friday, 2 May, as a public holiday created a four-day extended weekend (1–4 May), and the number of recorded visits increased from 249 on 24 April to 300 on 2 May. This temporal pattern is compatible with the hypothesis that reduced availability of primary care during extended holiday periods may contribute to increased ED utilization. However, causality cannot be established from the present descriptive analysis.

Previous research has emphasized the importance of calendar-related variables in forecasting ED attendance and has shown that temporal factors, particularly day-of-week effects, account for an important component of the observed variability in patient volume [10]. These findings support the consideration of calendar effects when interpreting short-term fluctuations in ED attendance and developing forecasting models.

Analysis of visits to the SCJU Craiova’s ED for the period January 2025–April 2026 reveals a distinct weekly pattern of patient attendance, characterized by statistically significant variations across days of the week. The descriptive statistics (Table 2), together with normality testing (Table 3) and non-parametric intergroup comparisons (Table 4), indicate systematic differences in ED attendance, with potential epidemiological and organizational implications.

In terms of average visit volume, Monday was the busiest day, with a mean of 257.6 patients/day, followed by Saturday (250.2), Sunday (247.37) and Friday (246.9). The lowest mean values were recorded during the Tuesday–Thursday interval, with relatively similar means (236.7, 234.1 and 233.5 patients/day, respectively). The descriptive pattern therefore indicates higher average attendance at the beginning of the week and during the Friday–Sunday interval, with comparatively lower attendance from Tuesday to Thursday. This pattern is consistent with previous research on patient flow in emergency care services [22] and may reflect differences in healthcare-seeking behavior and the accessibility of primary care and outpatient services across the week. The relatively higher attendance observed from Friday through Sunday may also be associated with factors reported in the literature, such as trauma, accidents, intoxications, and other socially determined emergencies [22]. However, these potential explanations were not directly evaluated in the present analysis.

Analysis of ED attendance variability revealed relatively low coefficients of variation across all days of the week, ranging from 8.08% on Monday to 12.44% on Wednesday. These values indicate relatively low dispersion of daily attendance around the corresponding weekday means. The lowest variability was observed on Monday (CV = 8.08%), suggesting that the relatively high Monday attendance was comparatively consistent across the observation period. From a managerial perspective, this degree of consistency may facilitate staffing estimation and shift planning. However, the descriptive statistics alone do not establish temporal predictability, which is subsequently addressed through the forecasting analysis.

The evaluation of distributional shape using skewness and kurtosis indicated distinct patterns across weekdays. The Monday distribution was approximately symmetric (skewness = −0.032), indicating little asymmetry in the distribution of daily visits. In contrast, Tuesday and Thursday exhibited positive skewness (0.992 and 0.744, respectively), indicating longer right tails and occasional days with relatively high attendance. Wednesday showed only slight positive skewness (0.207), whereas Friday and Saturday displayed moderate positive skewness (0.549 and 0.704, respectively). Sunday exhibited negative skewness (−1.030), indicating a pronounced left-tailed distribution, with most observations concentrated toward relatively higher attendance levels and a smaller number of lower-attendance days extending the distribution toward the lower end. The positive kurtosis observed for Sunday (0.912) indicates some departure from the reference normal distribution in terms of distributional shape. Overall, these descriptive characteristics indicate that weekday attendance distributions differ not only in their central tendency and variability but also in their distributional shape, providing additional justification for the subsequent normality assessment and non-parametric comparisons.

The Shapiro–Wilk test (Table 3) showed that the distributions for Monday, Wednesday, Friday and Sunday did not significantly deviate from normality (*p* > 0.05), whereas Tuesday, Thursday and Saturday exhibited non-normal distributions. Given the departures from normality observed in several weekday distributions, non-parametric methods were used for subsequent intergroup comparisons, including the Kruskal–Wallis test and pairwise Mann–Whitney U tests. From a practical perspective, the non-normality observed for Tuesday, Thursday and Saturday suggests that patient flow may occasionally be affected by episodic factors that generate asymmetric or non-Gaussian distributions.

Because the seven weekday groups exhibited departures from normality in several distributions, a non-parametric Kruskal–Wallis test was used to assess whether daily ED attendance differed across the seven days of the week. The analysis included all 485 daily observations from 1 January 2025 to 30 April 2026. The test revealed a statistically significant difference in ED attendance across weekdays, H(6) = 52.365, *p* < 0.001, with an epsilon-squared effect size of approximately 0.097. Thus, the null hypothesis of equal attendance distributions across the seven weekdays was rejected. This result indicates that the day of the week is associated with systematic differences in the distribution of daily ED presentations. However, the Kruskal–Wallis test does not identify which specific weekday pairs differ. 

To identify the specific weekday pairs contributing to the overall difference, pairwise Mann–Whitney U tests were subsequently performed with Holm’s correction for multiple comparisons (Table 4). 

The results demonstrate a statistically significant overall heterogeneity in daily ED attendance across the seven weekdays. The post hoc analysis indicates that the most pronounced and consistent differences involve Monday, which shows significantly higher attendance than Tuesday, Wednesday, Thursday and Friday after Holm adjustment. Additional significant differences were observed between selected mid-week and later-week days, particularly between Tuesday and Sunday, Wednesday and Saturday/Sunday, and Thursday and Saturday/Sunday. In contrast, no statistically significant differences were identified within the Tuesday–Thursday group or within the Friday–Sunday group after adjustment for multiple comparisons. These findings support the presence of a structured weekly pattern in ED attendance, with Monday representing a distinct high-attendance day, Tuesday–Thursday forming a relatively homogeneous lower-attendance interval and Friday–Sunday constituting a statistically less differentiated later-week interval. These patterns should be regarded as descriptive temporal regularities rather than evidence of distinct causal or operational regimes.

From an organizational perspective, the observed higher attendance on Mondays may have implications for staffing and operational capacity planning. In particular, the findings may support closer alignment of staffing levels, triage capacity, diagnostic resources, and observation-bed availability with the observed weekly demand pattern. During the weekend, staffing and resource allocation should likewise take into account the observed demand levels and the specific service requirements of emergency care. Conversely, the relatively lower and more homogeneous attendance observed during the Tuesday–Thursday interval may provide greater operational flexibility for administrative activities, staff training, and personnel scheduling, subject to service requirements.

From an epidemiological perspective, the observed weekly pattern indicates non-uniform healthcare-seeking behavior across the week. Differences in primary-care availability, delayed presentation, healthcare accessibility, and other social factors may contribute to this temporal variation; however, these potential explanations cannot be directly established from the present analysis.

### 4.2. Testing and Forecasting for SARIMA with Holiday Dummy Variable and Prophet Methods

SARIMA was selected as an interpretable statistical benchmark capable of explicitly modeling autocorrelation and the observed weekly seasonal structure, while Prophet was included as a complementary decomposition-based forecasting approach. The study therefore aimed at a focused comparison of two conceptually distinct forecasting frameworks rather than an exhaustive evaluation of all available forecasting algorithms.

To ensure a fair and consistent comparison between the SARIMA model with a holiday dummy variable and the Prophet model, both forecasting approaches were evaluated using a common rolling-origin (walk-forward) validation procedure. At each forecast origin, the models were estimated using all observations available up to that point and were subsequently used to generate forecasts over the same out-of-sample horizon. Thus, both models were evaluated on identical training and forecasting windows, eliminating differences arising from the use of different validation periods.

A forecast horizon of approximately 7–8 days was selected for the rolling-origin evaluation based on both operational considerations and the observed deterioration in SARIMA forecast performance at longer horizons. A one-week horizon is also directly relevant from an operational perspective, as it provides a sufficiently useful forecast window for short-term planning of emergency department resources while limiting the accumulation of forecast uncertainty at longer horizons.

The rolling-origin evaluation comprised four successive forecast origins:

Forecast origin 1: training period 1 January 2025–31 March 2026; forecasting period 1–7 April 2026.

Forecast origin 2: training period 1 January 2025–7 April 2026; forecasting period 8–15 April 2026.

Forecast origin 3: training period 1 January 2025–15 April 2026; forecasting period 16–22 April 2026.

Forecast origin 4: training period 1 January 2025–22 April 2026; forecasting period 23–30 April 2026.

Accordingly, the validation procedure used progressively expanding training samples while strictly preserving out-of-sample forecast evaluation at each origin. The forecast errors obtained across these common evaluation windows were subsequently used to compare the predictive performance of SARIMA with holiday dummy and Prophet using the selected accuracy measures.

Following the rolling-origin evaluation, both models were estimated using the complete available dataset up to 30 April 2026. The resulting final models were then used to generate the operational future forecast for 1–7 May 2026. This final forecast was not included in the calculation of predictive accuracy measures, because the corresponding observations were not available at the time of forecasting.

#### 4.2.1. Forecasting with the SARIMA Model with Holiday Dummy Variable

The analysis of the daily visits at SCJU Craiova’s ED between 1 January 2025 and 30 April 2026 was carried out using the SARIMA model with holiday dummy variable methodology, suitable for modeling chronological series characterized by serial dependence and periodic seasonality. SARIMA with holiday dummy variables was specified by extending the baseline SARIMA model with an exogenous binary indicator identifying Romanian legal holidays. The purpose of the analysis was to identify a stable temporal structure of addressability and build a robust predictive model for the forecast of the operational load of the ED. 

The dummy is

$${Holiday}_{t}=\{\begin{array}{cc} 1, & \;\;if\;day\;t\;is\;a\;legal\;holiday\\ 0, & otherwise \end{array}$$

The evaluation of the stationarity of the series was performed using the Augmented Dickey–Fuller (ADF) test (Table 5). The result (ADF = −4.6056; *p* = 0.0001) allowed the rejection of the null hypothesis, proving that the series is statistically stationary and does not require additional differentiation. This result indicates that the variations in daily visits fluctuate around a relatively stable mean and do not exhibit a deterministic long-term trend.

The analysis of the autocorrelation functions (ACF) and partial autocorrelation functions (PACF) (Table A1) highlighted the persistence of positive autocorrelations at small lags, as well as the appearance of recurrent peaks at multiples of lag 7, suggesting the existence of a strong weekly seasonality. The observed structure is consistent with the results of previous analyses on the variations in addressability depending on the day of the week and confirms the existence of a stable weekly operational rhythm of ED activity.

To identify the optimal model, several SARIMA with holiday dummy variable specifications were estimated and compared, using multiple statistical and diagnostic criteria: coefficient significance, Akaike [23] and Schwarz [24] information criteria, Durbin–Watson [21] statistics, Breusch–Godfrey tests for residual autocorrelation, Jarque–Bera test for normality and ARCH tests for conditional heteroscedasticity. Although the SARIMA(1,0,1)(0,0,1)_7_ model with a holiday dummy variable presented slightly more favorable values of the Akaike criterion (AIC = 9.263) and of the regression standard error, it revealed statistically significant residual serial autocorrelation in the Breusch–Godfrey test (*p* = 0.0001), indicating the existence of insufficiently modeled temporal dependencies. Consequently, the model considered optimal was SARIMA(1,0,5)(0,0,1)_7_ (Table 6), as it offered the best compromise between parsimony, stability and diagnostic validity.

**Figure 3:**
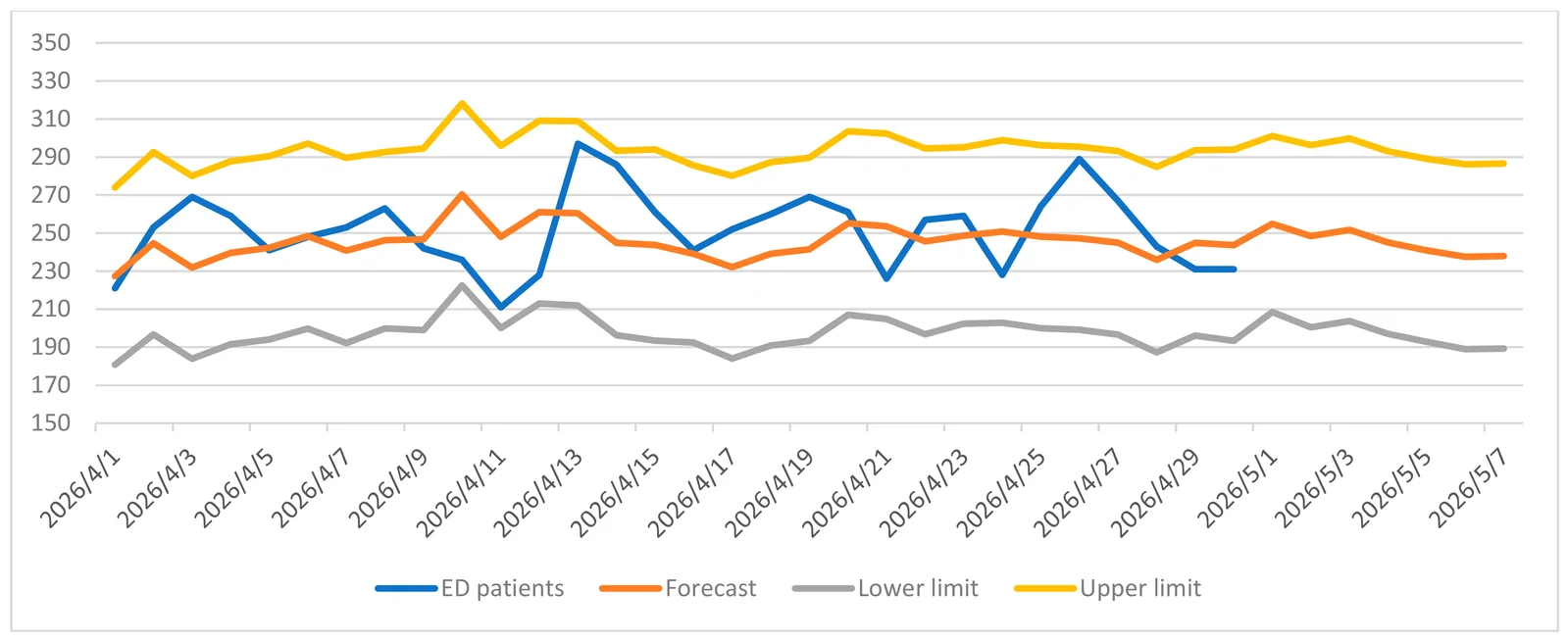
ED patients, forecast and confidence interval (SARIMA); authors’ contribution using Microsoft Excel, based on data from Table A5.

Thus, the model was tested for April 2026, using four periods for which the actual data are known, and the results are given in Table A2. It is found that the predictions are close to the actual values, the latter being within the confidence interval, at the 5% significance level. Also, the forecast for the first 7 days of May is shown in Table A2. The actual data, the forecast and the confidence interval are graphically represented in Figure 3.

All model coefficients were statistically significant: Holiday (*p* = 0.0005), AR(1) (*p* < 0.001), MA(5) (*p* = 0.0019), and SMA(7) (*p* < 0.001). The autoregressive AR(1) component indicates the presence of moderate temporal dependence between consecutive days, suggesting that the number of emergency department visits on a given day is partially influenced by the operational workload of the previous day. Compared with the SARIMA(1,0,1) model, in which the autoregressive coefficient was very high (AR = 0.922), the optimal model distributes temporal dependence more realistically across multiple lags through the inclusion of the MA(5) component, thereby reducing the excessive concentration of inertia within a single parameter.

The inclusion of the holiday dummy variable revealed a positive and statistically significant holiday effect. The estimated coefficient for HOLIDAY was 18.57 (t = 3.53, *p* < 0.001), indicating that legal holidays were associated with approximately 18.6 additional emergency department presentations compared with non-holiday days.

The MA(5) component indicates that recent model innovations, i.e., unexpected deviations between observed and model-implied attendance, contribute to the temporal dependence observed over several successive lags. This finding should not be interpreted as direct evidence of a five-day operational memory mechanism; rather, it reflects the short-term temporal dependence captured by the model through the recent history of forecast errors. From a substantive perspective, this pattern may be consistent with several possible mechanisms, including delayed healthcare-seeking, temporary accumulation of cases, constraints in access to primary care, or operational backlog following weekends and public holidays. However, these explanations remain plausible hypotheses rather than demonstrated mechanisms, as the corresponding epidemiological, healthcare-access, and organizational variables were not directly measured in the present study. Further research incorporating such covariates would be required to determine whether these factors contribute to the observed temporal dependence.

Model fit was considered satisfactory for a daily medical time series characterized by substantial biological and social variability. The model yielded a coefficient of determination of R^2^ = 0.247, a regression standard error of 24.1 and a Durbin–Watson statistic of 1.99, which is very close to the ideal value indicating residual independence. Although the R^2^ value is moderate, this result is regarded as acceptable in the context of daily epidemiological time series, which are influenced by numerous exogenous factors that are difficult to quantify, including epidemiological seasonality, weather conditions, accidents, accessibility of primary healthcare services and social or behavioral determinants.

Residual diagnostics confirmed the robustness of the model. The Jarque–Bera test has a value of 3.19, with the associated probability (*p*-value = 0.2019), indicating a normal distribution of the residuals. The Breusch–Godfrey test for serial autocorrelation produced a *p*-value of 0.576, indicating the absence of statistically significant residual autocorrelation at the conventional 5% significance level. This finding constitutes the primary methodological argument supporting the superiority of the SARIMA(1,0,5)(0,0,1)_7_ model over the SARIMA(1,0,1)(0,0,1)_7_ model, whose residuals retained substantial serial dependence (*p*-value = 0.0001). In practical terms, the residuals of the optimal model approximate a white-noise process, suggesting that the relevant systematic information has been adequately captured by the model structure.

The ARCH test for conditional heteroscedasticity revealed no significant effects (*p* = 0.64), demonstrating variance stability and the absence of volatility clustering. This result further supports the statistical robustness and predictive reliability of the model. 

Model performance was evaluated using RMSE and MAPE across the four common rolling-origin validation windows, while predictive uncertainty was assessed using 95% prediction intervals (Table 7).

The model’s out-of-sample forecasts for April 2026 showed a relatively stable level of predicted ED activity, with point forecasts ranging from 227.35 to 270.38 patients per day. The corresponding 95% prediction intervals were relatively wide, extending approximately from 180.73 to 318.26 patients, and therefore appropriately reflected uncertainty associated with short-term daily forecasting. Based on the 30 observations available for 1–30 April 2026, the model achieved an average RMSE of 21.75 patients and an average MAPE of 7.35%. According to the forecasting accuracy thresholds adopted in this study, a MAPE value below 10% indicates good predictive accuracy.

The model also captured relevant calendar effects during the Easter period. In particular, 10 April 2026 (Good Friday), 12 April 2026 (Easter Sunday) and 13 April 2026 (Easter Monday) were explicitly identified through the holiday dummy. The corresponding forecasts were 270.38, 261.04 and 260.46 patients, respectively. Although the model did not reproduce the observed values exactly, the inclusion of the holiday effect allows the forecasting framework to distinguish these dates from ordinary days and incorporates the systematic calendar information into the prediction process. This is particularly relevant for emergency department planning because healthcare-seeking behavior and the availability of alternative healthcare services may change during legal holidays. The higher forecasting errors observed in Origin 2 (8–15 April), with an RMSE of 30.16 patients and a MAPE of 11.03%, can plausibly be attributed to the Easter period in Romania, when ED attendance was higher and more variable than during the surrounding periods, despite the inclusion of the holiday dummy variable in the SARIMA specification.

Forecasts generated for 1–7 May 2026 predicted a relatively stable level of emergency department visits, ranging from approximately 238 to 255 patients per day. The corresponding 95% prediction intervals ranged from approximately 189 to 301 patients, indicating a moderate level of forecast uncertainty and a relatively stable short-term pattern of patient flow. The model captured the previously observed weekly seasonal pattern while explicitly incorporating the effect of legal holidays through the holiday dummy variable. In particular, the forecast for 1 May 2026, a legal holiday in Romania, was 254.81 patients, compared with forecasts of approximately 238–252 patients for the remaining days of the forecast horizon.

From an epidemiological and organizational perspective, these findings suggest that activity at SCJU Craiova’s ED is not entirely random but is influenced by systematic autoregressive, moving-average, weekly seasonal and calendar-related mechanisms. The statistically significant holiday effect indicates that legal holidays are associated with an estimated increase of approximately 18.6 ED presentations, ceteris paribus. These findings support the implementation of adaptive medical resource planning strategies, particularly during legal holidays and periods with predictable changes in emergency department demand, as well as the continuous monitoring of workload throughout the week.

#### 4.2.2. Forecasting with the Prophet Method

Forecasting was performed using the Prophet time-series model without yearly seasonality, implemented in Python. Model performance was evaluated using RMSE and MAPE on the test dataset, while prediction uncertainty was assessed through 95% confidence intervals. Algorithm 1 is as follows:
**Algorithm 1** Prophet time-series model, implemented in Python  import pandas as pd   from prophet import Prophet   import matplotlib.pyplot as plt   import numpy as np   import pandas as pd   from prophet import Prophet   import matplotlib.pyplot as plt   # Loading datasheet   # Forecast origin 1   # Prophet training: 1 January 2025–31 March 2026   # Prophet forecast: 1–7 April 2026—7 days forecast   # Forecast origin 2   # Prophet training: 1 January 2025–7 April 2026   # Prophet forecast: 8–15 April 2026—8 days forecast   # Forecast origin 3   # Prophet training: 1 January 2025–15 April 2026   # Prophet forecast: 16–22 April 2026—7 days forecast   # Forecast origin 4   # Prophet training: 1 January 2025–22 April 2026   # Prophet forecast: 23–30 April 2026—8 days forecast   # Forecast origin 5   # Prophet training: 1 January 2025–30 April 2026   # Prophet forecast: 1–7 May 2026—7 days forecast   #(First training dataset)   df = pd.read_csv(‘DataSheet_01.01.25_31.03.26.csv’, sep = ‘;’)   df = df.rename(columns = {‘Date’: ‘ds’, ‘Number of emergency patients’: ‘y’})  df[‘ds’] = pd.to_datetime(df[‘ds’], format = ‘%d.%m.%Y’)   # Configuring the model with holidays in Romania   model = Prophet(growth = ‘flat’,yearly_seasonality = False, weekly_seasonality = True, interval_width = 0.95, uncertainty_samples = 5000)   model.add_country_holidays(country_name = ‘RO’)  model.fit(df)   # Generating the 7-day forecast (or 8-day forecast, depending on the period)   future = model.make_future_dataframe(periods = 7)   #for 8-day forecast parameter periods = 8   forecast = model.predict(future)   # Results visualization   model.plot(forecast)   plt.title(“ED Patient Flow Forecast Craiova”)  plt.show()   # View components (Trend, Holidays, Seasonality)  model.plot_components(forecast)  plt.show()   # Display the date, point forecast and confidence interval bounds  #.tail(7) displays the last 7 rows (the most recent forecasts)  display(forecast[[‘ds’, ‘yhat’, ‘yhat_lower’, ‘yhat_upper’]].tail(7))   # Generate forecast results for 1st period, each training interval will have a corresponding forecast results CVS file.   forecast[[‘ds’, ‘yhat’, ‘yhat_lower’, ‘yhat_upper’]].to_csv(‘forecast_upu_craiova2026_01_07_april.csv’, index = False)

The model was tested for April 2026, using four periods as in the SARIMA case, and the results are given in Table A3. It is found that the predictions are very close to the real values, the latter being within the confidence interval at a significance threshold of 5%. The forecast for 1–7 May is given in Table A3. 

**Figure 4:**
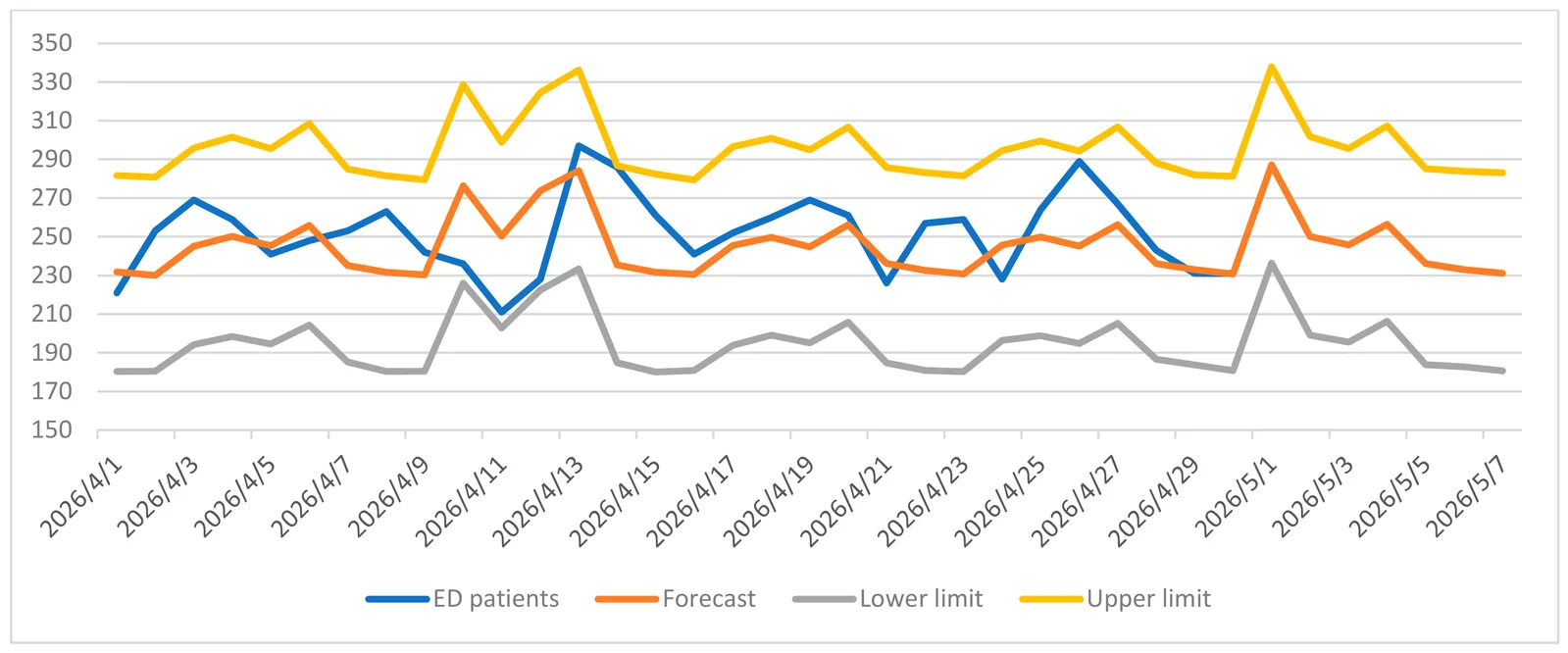
ED patients, forecast and confidence interval (Prophet), authors’ contribution with Microsoft Excel, based on data from Table A5.

The actual data for April, the forecasts for April and 1–7 May and the confidence intervals are graphically represented in Figure 4.

Model performance was evaluated using RMSE and MAPE across the four commonly used rolling-origin validation windows. Prophet achieved an average RMSE of 21.64 patients and an average MAPE of 7.43% (Table 8).

Forecasts generated for 1–7 May 2026 using the Prophet model without yearly seasonality predicted a relatively stable level of emergency department visits, ranging from approximately 231 to 287 patients per day. The highest forecast was recorded for 1 May (287.12 patients), which is a Romanian legal holiday and is explicitly accounted for in the Prophet model through the country-specific holiday component. Forecasts for the subsequent days ranged between approximately 231 and 256 patients, indicating a relatively stable expected workload after the holiday peak. The 95% prediction intervals reflected the uncertainty associated with short-term daily forecasts.

The rolling-origin evaluation indicated good overall forecasting performance, with an average RMSE of 21.64 patients and an average MAPE of 7.43%. The highest forecasting errors occurred in Origin 2 (8–15 April), with an RMSE of 35.30 patients and a MAPE of 13.22%. This higher error is plausibly related to the Easter period in Romania, when ED attendance was higher and more variable than during ordinary periods. Although Prophet explicitly incorporates Romanian holidays, the magnitude and temporal dynamics of the Easter-related increase may not have been fully captured by the model.

Overall, the Prophet model without yearly seasonality demonstrates good short-term forecasting performance while accounting for Romanian legal holidays. The results suggest that incorporating calendar effects improves the representation of atypical periods, although exceptional events such as the Easter period may still generate larger forecasting errors due to unusually high and variable ED demand.

### 4.3. Comparative Evaluation of SARIMA and Prophet Models

The predictive performance of the SARIMA and Prophet models was assessed using a common rolling-origin, out-of-sample validation framework. Both models were evaluated over the same four successive forecast windows covering 1–30 April 2026, with identical observed outcomes and forecasting horizons (Table 9). The comparison was based on the Root Mean Square Error (RMSE) and Mean Absolute Percentage Error (MAPE). In addition, aggregate RMSE and MAPE were calculated over the complete 1–30 April 2026 evaluation period, and the Diebold–Mariano (DM) test with a HAC/Newey–West correction was employed to assess whether the observed differences in predictive accuracy were statistically significant.

The SARIMA specification used in the comparative analysis was the selected SARIMA(1,0,5)(0,0,1)_7_ model with a holiday dummy variable, while Prophet was specified with Romanian holiday effects, weekly seasonality, and without yearly seasonality. Both models were estimated using information available only up to the corresponding forecast origin, thereby ensuring that the April observations used for validation were genuinely out-of-sample.

The results indicate that the relative performance of the two models varied across the four forecast origins. Prophet obtained lower RMSE values in Origins 1, 3 and 4, whereas SARIMA performed better in Origin 2. A similar pattern was observed for MAPE: Prophet achieved lower values in Origins 3 and 4, while SARIMA performed better in Origins 1 and 2. Therefore, neither model consistently dominated the other across all validation windows.

The largest forecasting errors for both models occurred in Origin 2 (8–15 April 2026). SARIMA recorded an RMSE of 30.16 and MAPE of 11.03%, whereas Prophet recorded an RMSE of 35.30 and MAPE of 13.22%. This deterioration in predictive accuracy was plausibly related to the unusual attendance pattern during the Easter period in Romania. In particular, the observed number of ED presentations during this period differed substantially from the usual temporal pattern, creating a more challenging forecasting situation for both models. The presence of such an atypical calendar-related episode illustrates the difficulty of forecasting short-term emergency department demand around major public holidays, even when holiday information is incorporated into the models.

In Origin 1, SARIMA achieved a lower MAPE than Prophet (4.70% vs. 5.48%), although Prophet had a slightly lower RMSE (15.61 vs. 16.93). In Origin 3, Prophet clearly outperformed SARIMA, with an RMSE of 15.00 compared with 18.96 and a MAPE of 5.13% compared with 6.54%. In Origin 4, the difference was smaller, with Prophet again showing slightly lower RMSE and MAPE values. These results demonstrate that model performance was sensitive to the particular temporal characteristics of each validation window rather than being systematically determined by the model class.

#### 4.3.1. Aggregate Evaluation over the Complete April 2026 Period

To complement the rolling-origin analysis, RMSE and MAPE were also calculated directly over the entire 1–30 April 2026 out-of-sample period (Table 10). This approach avoids assigning equal weight to the four validation windows and provides a single measure of overall predictive performance based on all 30 daily observations.

The aggregate results provide an important complementary perspective. Over the complete April 2026 evaluation period, SARIMA achieved slightly lower forecasting errors than Prophet, with an RMSE of 22.59 compared with 23.54 and a MAPE of 7.46% compared with 7.56%. The differences are nevertheless very small: approximately 0.95 patients for RMSE and 0.10 percentage points for MAPE.

Thus, the aggregate evaluation does not indicate a substantial practical difference in predictive accuracy between the two approaches. Rather, it suggests that the two models provide broadly comparable short-term forecasts, with a marginal numerical advantage for SARIMA when the entire April evaluation period is considered.

#### 4.3.2. Diebold–Mariano Test with HAC/Newey-West Correction

Because differences in RMSE and MAPE alone do not establish whether one forecasting model is statistically more accurate than another, the Diebold–Mariano test (Table 11) was applied to the daily forecast errors over the entire 1–30 April 2026 period. The squared-error loss function was used:

$$d_{t}=e_{{SARIMA}_{t}}^{2}-e_{{Prophet}_{t}}^{2}$$

where $e_{{SARIMA}_{t}}^{2}$ and $e_{{Prophet}_{t}}^{2}$ denote the forecast errors of the two models. Under this formulation, a negative value of the mean loss differential indicates a numerical advantage for SARIMA.

Given the daily nature of the observations and the possibility of serial dependence in forecast-error losses, the variance of the loss differential was estimated using a HAC/Newey–West correction. A lag of one was used as the primary specification, with additional lag lengths used as a robustness check.

The negative mean loss differential indicates that SARIMA had a lower average squared forecasting error over the evaluation period, consistent with its slightly lower aggregate RMSE. However, the Diebold–Mariano statistic was −0.567 with a *p*-value of 0.575, indicating that the difference was not statistically significant. Consequently, the null hypothesis of equal predictive accuracy cannot be rejected.

The robustness analysis using alternative Newey–West lag lengths led to the same conclusion (Table A4). Across all HAC lag specifications considered, the *p*-value remained substantially above the 5% significance threshold. Therefore, the conclusion is not sensitive to the choice of the Newey–West lag within the range examined. Although the numerical sign of the DM statistic consistently favors SARIMA, the magnitude of the difference is insufficient to establish statistically superior predictive performance.

#### 4.3.3. Interpretation of the Comparative Results

The comparison therefore provides a more nuanced picture than the individual RMSE and MAPE values might suggest. Prophet achieved lower errors in several individual rolling-origin windows, particularly in Origins 3 and 4, whereas SARIMA performed better in Origins 1 and 2. The second validation window represented a particularly challenging period for both models because it included the Easter period in Romania, during which ED attendance exhibited atypical behavior. The higher RMSE and MAPE observed during this period consequently reflect, at least in part, the difficulty of forecasting emergency department demand during an unusual holiday-related episode.

The aggregate evaluation over all 30 days slightly favored SARIMA. Its RMSE was 22.59 patients and its MAPE was 7.46%, compared with 23.54 patients and 7.56% for Prophet. Nevertheless, the Diebold–Mariano test showed that this difference was not statistically significant. Therefore, the results do not provide evidence that either model has statistically superior predictive accuracy over the entire April 2026 evaluation period.

An additional aspect of the comparison concerns the treatment of calendar effects. Both models incorporated Romanian holiday information, but they represented these effects differently. SARIMA incorporated holidays through an explicit dummy variable, whereas Prophet included the Romanian holiday calendar as an additional model component. This common inclusion of holiday information makes the comparison more balanced than a specification in which only one model accounts for calendar effects.

The final forecasts for 1–7 May 2026 further illustrate the different forecasting profiles of the two approaches. SARIMA generated forecasts ranging from 237.60 to 254.81 patients per day, while Prophet produced forecasts between 231.11 and 287.12 patients per day. The particularly high Prophet forecast of 287.12 patients on 1 May reflects the incorporation of the 1 May public holiday effect, whereas SARIMA also produces an elevated forecast of 254.81 patients for the same date. Thus, both models recognize the calendar effect, although its magnitude differs between the two approaches.

The SARIMA forecasts display a relatively smoother temporal profile, consistent with the model’s explicit representation of autoregressive, moving-average and weekly seasonal dependence. Prophet produces greater day-to-day variation, reflecting its additive representation of weekly seasonality and holiday effects. These differences are relevant from an operational perspective because a model with lower error does not necessarily provide the same representation of the temporal dynamics of ED attendance.

## 5. Discussions

### 5.1. Weekly Structure and Temporal Dependence

The analysis of ED attendance at SCJU Craiova revealed a structured pattern of variation across the days of the week. Monday recorded the highest mean attendance (257.6 patients/day), and the post hoc analysis showed statistically significant differences between Monday and Tuesday, Wednesday, Thursday, and Friday after Holm adjustment. However, differences between Monday and Saturday or Sunday were not statistically significant. This pattern is broadly consistent with the recurrent weekly variations reported in previous ED forecasting research [3], while the present results provide a more specific characterization of which weekday comparisons remain statistically supported after adjustment for multiple testing. The findings therefore indicate a non-uniform weekly distribution of ED attendance rather than a uniformly elevated Monday effect across all other days.

The observed weekly structure was further supported by the time-series analysis. The ACF/PACF results and the significant seasonal moving-average component in the SARIMA(1,0,5)(0,0,1)_7_ model indicate that observations separated by seven days contain relevant predictive information. This finding is in line with previous applications of seasonal time-series models to healthcare demand [4] and indicates that weekly temporal dependence is an important characteristic of the Craiova ED attendance series. The result also provides a statistical basis for incorporating weekly seasonality into short-term forecasting rather than treating daily attendance as an independent sequence of observations.

These findings should nevertheless be distinguished from ED overcrowding. The present study identified recurrent differences in the volume of patient presentations but did not directly measure the relationship between demand and available ED capacity, waiting times, length of stay, or other indicators of overcrowding. Consequently, the higher attendance observed on particular days should not be interpreted as evidence of overcrowding. In the context of the literature on ED overcrowding [2], the present findings are better understood as identifying temporal variations in demand that may be operationally relevant for anticipating changes in workload.

The results may also be considered in relation to the operational consequences associated with ED overcrowding [15]. Although the present analysis cannot establish whether the higher-attendance periods generated treatment delays or capacity constraints, identifying recurrent variations in patient volume provides an earlier indication of when demand is more likely to increase. Such information may be useful for forward-looking resource planning, while the actual effect of forecast-informed staffing or capacity adjustments on waiting times and patient flow would require separate operational evaluation.

### 5.2. Comparison with Previous Forecasting Studies

The findings concerning weekly variation are also comparable with the results reported by Whitt and Zhang [7]. In the present study, the statistically significant weekday differences and the detected weekly dependence indicate that the calendar position of a day contains relevant information for explaining short-term variation in ED attendance. At the same time, the present analysis differs in its emphasis on the temporal structure of daily attendance and in its use of a common out-of-sample forecasting framework. This suggests that meaningful short-term information can be obtained from the historical dynamics of ED attendance itself, while additional external variables may represent potential extensions of the forecasting framework.

The comparison between SARIMA and Prophet provides further insight into the forecasting component of the study. Both models showed good short-term predictive performance across the four common rolling-origin evaluation windows, but their relative performance varied across individual periods. Prophet performed better in some windows, particularly Origins 3 and 4, whereas SARIMA performed better in Origins 1 and 2. The Easter period represented a particularly challenging forecasting interval for both models, illustrating the difficulty of predicting attendance during an atypical holiday-related episode. 

When the complete April 2026 evaluation period was considered, SARIMA produced slightly lower aggregate errors than Prophet (RMSE 22.59 vs. 23.54 patients; MAPE 7.46% vs. 7.56%). However, the Diebold–Mariano test did not indicate a statistically significant difference in predictive accuracy. Therefore, the numerical differences in RMSE and MAPE should not be interpreted as evidence of model superiority. Rather, the results indicate that SARIMA and Prophet provided statistically comparable forecasting accuracy under the common out-of-sample evaluation design.

The findings can be interpreted alongside previous applications of Prophet and SARIMA in healthcare forecasting [10,11]. The present results do not reproduce a clear advantage for either model as reported in those studies, but instead suggest that their relative performance may depend on the characteristics of the specific forecasting problem. In particular, differences in the outcome variable, temporal aggregation, forecasting horizon, seasonal structure, and information available to the models may influence predictive performance. The comparable results obtained here therefore support an empirical approach to model selection in which competing methods are evaluated under identical out-of-sample conditions rather than assuming that one forecasting framework will consistently outperform another.

A quantitative comparison with previously published results requires attention to the outcome variable and the level of temporal aggregation, since these largely determine the attainable level of accuracy. Against a mean daily attendance of 243.72 patients, the aggregate MAPE obtained here (7.46% for SARIMA and 7.56% for Prophet) corresponds to a mean absolute error of approximately 18 patients per day, while the aggregate RMSE of 22.59 patients represents 9.3% of mean daily attendance. These values are of the same order as those reported by Whitt and Zhang [7], whose best-performing SARIMAX specification achieved a test-set MAPE of 8.4% for daily arrivals, and are obtained here without recourse to meteorological covariates. Considerably lower error rates have been reported elsewhere, but for outcomes and aggregations that are intrinsically smoother: Fattouh et al. [10] obtained MAPE values between 3.21% and 3.53% for hospital bed occupancy, a stock variable that changes gradually because it is constrained by admission and discharge processes, while Chen and Zhou [11] modeled monthly infection counts, an aggregation at which much of the day-to-day noise present in the current series is averaged out. Daily arrival counts are considerably more volatile than either, and the residual variability accordingly remains high: the selected SARIMA specification explains 24.7% of the variance in daily attendance, which is consistent with the range typically reported for daily ED arrival series and reflects the substantial component of demand that is driven by factors not observable from attendance data alone. The comparison therefore indicates that the accuracy obtained here is appropriate to the forecasting problem addressed, rather than deficient relative to studies that address a different one.

Two further aspects of the results find direct support in the recent literature. First, the concentration of forecast error in the second validation window is consistent with the findings of Rostami-Tabar and Ziel [13], who demonstrated that public holidays and other special events constitute a distinct and disproportionate source of error in emergency department forecasting, and that the manner in which such effects are represented has a material influence on predictive accuracy. In the present study, both models incorporated the Romanian holiday calendar, and both nevertheless produced their largest errors during the Easter window, with the SARIMA holiday coefficient of 18.57 patients evidently understating the magnitude of the observed Easter-related deviation. This suggests that a single binary holiday indicator, or an undifferentiated holiday component, may be insufficient for holidays whose effects extend over several days and differ in magnitude between one holiday and another. Second, the absence of a statistically significant difference between the two approaches is consistent with a broader pattern in recent comparative work. Sudarshan et al. [8] found that no single method dominated across the forecasting configurations they examined, and Vollmer et al. [12] similarly reported that machine-learning approaches did not consistently outperform well-specified statistical benchmarks for emergency department demand. The present result extends this pattern to a formal test of equal predictive accuracy applied under strictly equivalent out-of-sample conditions, and supports the view expressed in the review of Gul and Celik [6] that model selection in this domain is best resolved empirically for the specific forecasting problem at hand rather than by general preference for a model class.

An additional consideration concerns the representation of calendar effects. In the present study, both SARIMA and Prophet incorporated Romanian holiday information, although through different modeling structures. This common information set provides a more balanced basis for comparison and is particularly relevant for the interpretation of the forecasts around holiday periods. The models nevertheless produced somewhat different forecast profiles: SARIMA generated smoother predictions, whereas Prophet showed greater day-to-day variation. These differences indicate that forecasting accuracy alone does not fully characterize the practical behavior of a forecasting model, particularly when forecasts are intended to inform short-term operational planning.

The present findings also complement the broader literature on predictive approaches in emergency care [1,5]. Rather than evaluating downstream operational outcomes directly, this study focuses on the earlier stage of anticipating daily patient attendance. The recurrent temporal structure identified in the data and the relatively stable predictive performance across successive out-of-sample windows indicate that routinely collected historical attendance data contain information that can be used to anticipate near-term changes in patient volume. This provides a potentially useful evidence base for forward-looking planning, although the extent to which forecast information translates into measurable improvements in staffing, waiting times, patient flow, or resource utilization remains to be established empirically.

### 5.3. Methodological Positioning Relative to Previous Approaches

The contribution of the present study lies in the design of the comparative evaluation rather than in any claim of superior forecasting accuracy. The Diebold–Mariano test reported above found no statistically significant difference in predictive accuracy even between the two models compared here, and we therefore make no claim that either model forecasts emergency department attendance more accurately than those employed in previous work. What the present design does address are four limitations that recur in the applied literature on emergency department demand forecasting.

The first concerns the equality of the information available to competing models. Comparisons between Prophet and classical seasonal models are frequently conducted with the holiday calendar supplied to Prophet, in which it is an integral component of the specification, while the competing SARIMA model is estimated as a purely univariate series. Any resulting difference in accuracy then reflects, at least in part, the presence or absence of calendar information rather than the properties of the model classes themselves. In the present study both models received equivalent calendar information: SARIMA through an explicit Romanian legal-holiday dummy variable, whose estimated coefficient of 18.57 patients is directly interpretable, and Prophet through the country-specific holiday component. The comparison is therefore between two representations of the same information rather than between an informed and an uninformed specification.

The second concerns the structure of the out-of-sample evaluation. A substantial proportion of published studies assesses forecasting accuracy on a single fixed train–test partition. Tashman [25] demonstrated that evaluations of this kind are sensitive to the particular characteristics of the selected test period and that rolling-origin designs, in which the forecast origin is advanced successively and forecasts are generated afresh from each origin, yield more reliable estimates of out-of-sample accuracy. The relevance of this consideration is evident in the present results: had the evaluation been confined to a single window, the conclusion would have depended materially on which window was chosen, since Prophet achieved the lower RMSE in Origins 1, 3 and 4 while SARIMA performed better in Origin 2. Four successive origins with identical horizons and identical observed outcomes were therefore employed for both models.

The third concerns the basis on which conclusions about relative performance are drawn. Differences in RMSE and MAPE are point estimates subject to sampling variation, and a numerical difference between two such values does not establish that the underlying predictive accuracies differ. The review of Gul and Celik [6] indicates that formal comparison of predictive accuracy remains uncommon in this literature, with conclusions frequently resting on the ordering of reported error measures alone. In the present study the difference in aggregate RMSE between the two models was 0.95 patients, corresponding to 0.4% of mean daily attendance; a comparison based on point estimates alone would have reported an advantage for SARIMA. The Diebold–Mariano test with HAC/Newey–West correction shows that a difference of this magnitude cannot be distinguished from sampling variation, and the sensitivity analysis across alternative lag specifications reported in Table A4 confirms that this conclusion does not depend on the choice of bandwidth.

The fourth concerns the separation of distributional analysis from forecasting. Studies that characterize the temporal structure of attendance and studies that construct forecasting models are typically distinct, with the consequence that the structure identified statistically and the structure exploited predictively are not formally connected. The present framework integrates the two: the weekly variation established through the Kruskal–Wallis test and the Holm-corrected post hoc comparisons is the same structure subsequently captured by the seasonal moving-average component of the SARIMA specification and by the weekly seasonality of the Prophet model, so that the inferential and predictive components of the analysis address a common object.

One limitation of the present design should be stated alongside these considerations. The comparative evaluation rests on thirty daily observations, which are few for a test of equal predictive accuracy. Harvey et al. [26] showed that the standard Diebold–Mariano statistic is oversized in samples of this magnitude and is therefore disposed towards rejection of the null hypothesis of equal accuracy. Since the test did not reject that null here, this property renders the conclusion conservative rather than a consequence of insufficient power; a small-sample correction of the kind proposed by those authors would move the *p*-value further from the conventional significance threshold. Extension of the evaluation window remains desirable nonetheless and is identified among the directions for future research.

### 5.4. Complementary Role of Simulation-Based Approaches

Beyond forecasting, simulation-based approaches provide an important complementary perspective for emergency department management. Discrete Event Simulation (DES) can represent patient arrivals, queues, service processes, and resource constraints, allowing alternative operational configurations to be evaluated without modifying the real system [27,28]. Recent applications have used DES to examine patient flow and alternative operational configurations in emergency departments, while DES–DOE frameworks have extended this approach to the systematic evaluation of operational factors and scenarios in healthcare settings [28,29]. In this context, the forecasting models evaluated in the present study should not be interpreted as substitutes for simulation or optimization approaches. Rather, short-term demand forecasts could serve as inputs to future DES-based analyses, enabling predicted patient arrivals to be combined with information on capacity, staffing, service times, and patient flow.

From an operational perspective, the results therefore support a cautious interpretation of forecasting as a decision-support instrument rather than an optimization mechanism in itself. The identification of recurrent high- and low-attendance periods can inform planning of workforce availability and other ED resources, but the present study does not test whether implementing such adjustments would improve operational performance. Future research could build on these forecasts by integrating predicted attendance with capacity, staffing, waiting-time, and patient-flow data, including through simulation or optimization approaches.

### 5.5. Practical Implications

Overall, the findings indicate that daily ED attendance at SCJU Craiova contains a stable weekly structure and sufficient temporal information to support short-term forecasting. The statistical analysis identifies specific weekday differences, while the forecasting analysis demonstrates that both SARIMA and Prophet can provide useful predictions under a common out-of-sample framework. Importantly, the absence of a statistically significant difference in predictive accuracy between the two models suggests that the principal contribution of the forecasting component lies in demonstrating the predictability of ED attendance rather than establishing a universally superior forecasting method.

From a practical perspective, the magnitudes involved can be expressed in terms directly relevant to operational planning. The difference between the highest- and lowest-attendance weekdays, 257.60 patients on Monday against 233.50 on Thursday, amounts to 24.1 patients per day, or 9.9% of mean daily attendance; the estimated effect of a legal holiday, 18.57 additional presentations, is of a comparable order at 7.6%. Differentials of this size are recurrent and identifiable in advance, and are therefore amenable to anticipatory rather than reactive adjustment of staffing and treatment-area capacity. The forecast error must, however, be interpreted alongside them: an RMSE of approximately 22.6 patients means that the expected deviation of a daily forecast is of a similar magnitude to the weekday differential itself. The forecasts are consequently suited to informing decisions at the level of the week—the ordering of staffing intensity across weekdays and the identification of holiday periods requiring augmented cover—rather than to determining precise daily staffing establishments, for which the prediction intervals reported in Table A2 and Table A3 constitute the appropriate basis for planning. Finally, the absence of a statistically significant difference in predictive accuracy between the two models carries a practical implication of its own: since neither approach can be preferred on grounds of accuracy, selection between them may reasonably be governed by considerations of implementation. SARIMA offers explicitly interpretable coefficients, including a directly quantified holiday effect, which may be advantageous where the model is intended to support discussion with clinical and administrative staff, whereas Prophet requires less specification effort and accommodates missing observations and structural change more readily, which may be preferable where forecasts are to be regenerated routinely with limited analytical supervision.

### 5.6. Study Limitations

This study has several limitations. First, it was conducted at a single emergency department, the Craiova County Emergency Clinical Hospital (SCJU Craiova), which may limit the generalizability of the findings to other healthcare institutions. Second, although the January 2025–April 2026 observation period was sufficient to identify weekly seasonality and short-term serial dependence, it does not allow for the assessment of longer-term or inter-annual patterns.

The forecasting models were based primarily on aggregated daily ED attendance and did not incorporate potentially relevant exogenous variables, such as weather conditions, epidemiological indicators, local events, or healthcare accessibility. In addition, the analysis did not stratify patients by triage category, diagnosis, age, or clinical severity, limiting the ability to identify potentially different demand patterns across patient groups.

The study assessed ED attendance rather than overcrowding directly. Therefore, the forecasts should not be interpreted as direct measures of overcrowding, which also depends on staffing, bed availability, length of stay, and patient-flow processes. Finally, although SARIMA and Prophet were compared using a common rolling-origin out-of-sample framework, the comparative validation covered only 1–30 April 2026. Consequently, the findings regarding their relative predictive accuracy should be interpreted within the specific validation period examined.

### 5.7. Future Research Directions

Future research should extend the analysis to multiple emergency departments and longer observation periods, allowing assessment of the generalizability and temporal stability of the identified patterns. Incorporating exogenous variables, including weather, epidemiological indicators, local events, and healthcare accessibility, could further improve forecasting performance.

Further studies should also consider clinical stratification by triage category, diagnosis, age, and hospitalization requirement, enabling more targeted forecasts and resource allocation. Comparative analyses could additionally evaluate machine-learning and hybrid approaches, such as LSTM and SARIMA–machine-learning models, using the same rigorous out-of-sample validation framework.

Finally, future research could link ED attendance forecasts with operational indicators such as staffing, bed occupancy, waiting times, and length of stay. This would allow forecasting to move beyond patient volume toward operational demand prediction and more precise emergency-care resource planning.

## 6. Conclusions

The present study analyzed the dynamics of emergency department (ED) attendance at the Craiova County Emergency Clinical Hospital between January 2025 and April 2026 using an integrative analytical framework combining descriptive statistics, nonparametric hypothesis testing, and time-series forecasting through SARIMA and Prophet. The results demonstrate that daily ED attendance exhibits a structured temporal pattern characterized by statistically significant differences across weekdays, recurrent weekly seasonality, and serial dependence. The combined use of the Kruskal–Wallis test and post hoc Mann–Whitney U tests with Holm correction provided evidence of significant differences between specific weekday distributions. The time-series analysis further identified a recurrent seven-day seasonal component and short-term serial dependence in daily ED attendance. Both SARIMA and Prophet demonstrated useful out-of-sample forecasting performance under a common rolling-origin validation framework. Although Prophet generally produced lower numerical prediction errors, the Diebold–Mariano test with HAC/Newey–West correction did not identify a statistically significant difference in predictive accuracy between the two approaches.

### Testing the Research Hypotheses Provided Evidence Supporting the Statistical and Forecasting Objectives of the Study

**H1:**  *The Kruskal–Wallis test identified statistically significant differences in ED attendance across the seven weekdays, with H(6) = 52.365 and p < 0.001. This result indicates that the null hypothesis of identical attendance distributions across weekdays can be rejected. Therefore, Hypothesis H1 was supported*.

**H2:**  *Following the statistically significant Kruskal–Wallis result, post hoc pairwise comparisons were performed using the Mann–Whitney U test with Holm correction for multiple comparisons. These analyses identified specific weekday pairs for which statistically significant differences in ED attendance remained after adjustment for multiple testing. The post hoc results therefore provide evidence that the overall weekday effect identified by the Kruskal–Wallis test is attributable to specific differences between weekday distributions. Consequently, Hypothesis H2 was supported*.

**H3:**  *Analysis of the autocorrelation function (ACF) and partial autocorrelation function (PACF) revealed persistent serial dependence and recurring peaks at lag 7 and its multiples, indicating a stable weekly seasonal component in daily ED attendance. The selected SARIMA(1,0,5)(0,0,1)_7_ model included statistically significant AR(1), MA(5), and seasonal MA(7) components. The significant seasonal component is consistent with a recurrent seven-day pattern, while the MA(5) term indicates that recent model innovations contribute to the short-term temporal dependence of the attendance series. Importantly, the MA(5) component is not interpreted as direct evidence of a five-day operational memory or as proof of a specific epidemiological or organizational mechanism. On this basis, Hypothesis H3 was supported*.

**H4:**  *The forecasting models demonstrated satisfactory out-of-sample predictive performance under the common rolling-origin evaluation framework. Across the four successive forecasting windows, both SARIMA and Prophet produced relatively low prediction errors, although the magnitude of the errors varied across forecast origins. Both models were evaluated on identical forecast periods, observed outcomes, forecasting horizons, and accuracy measures. The results demonstrate that daily ED attendance can be forecast with useful short-term accuracy using time-series approaches under an out-of-sample validation design. Therefore, Hypothesis H4 was supported*.

**H5:**  *The comparative evaluation showed that neither SARIMA nor Prophet consistently outperformed the other across the rolling-origin validation windows. Although SARIMA showed a marginal numerical advantage over the entire April 2026 evaluation period, the Diebold–Mariano test indicated no statistically significant difference in predictive accuracy (p = 0.575). Therefore, H5 was supported, indicating comparable predictive accuracy between the two models*.

From a managerial perspective, the findings support the use of either forecasting approach for short-term ED attendance planning, depending on the intended analytical purpose. The relatively similar predictive accuracy of the two models suggests that both can provide useful information for workforce scheduling, resource allocation, and patient-flow planning. The identification of weekly attendance patterns and holiday-related variations may further assist healthcare managers in anticipating periods of potentially increased or decreased demand. In particular, the forecasts for 1–7 May 2026 illustrate that both models recognize the effect associated with the 1 May public holiday, although they estimate its magnitude differently. However, the present study directly analyzes ED attendance and demand rather than ED overcrowding itself. Consequently, the findings should not be interpreted as a direct measurement of overcrowding, which is a multidimensional phenomenon involving the interaction between demand and system capacity, including staffing, bed availability, length of stay, treatment delays, and patient-flow processes. Nevertheless, improved anticipation of fluctuations in ED demand may contribute to operational preparedness during periods when demand places increased pressure on available emergency-care capacity.

From a scientific perspective, this study contributes to the literature on the statistical analysis and forecasting of emergency healthcare demand in Romania by integrating distributional assessment, nonparametric hypothesis testing, multiple-comparison correction, and complementary time-series forecasting approaches within a single analytical framework. The combination of the Kruskal–Wallis test, post hoc Mann–Whitney U tests with Holm correction, SARIMA, and Prophet provides a comprehensive framework for examining both the distributional differences and temporal dynamics of daily ED attendance. The use of a common rolling-origin, out-of-sample validation framework, complemented by aggregate accuracy measures and a formal Diebold–Mariano test with HAC/Newey–West correction, strengthens the methodological basis for comparing the two forecasting approaches.

The comparative analysis also highlights an important distinction between numerical forecasting accuracy and statistically significant model superiority. The individual rolling-origin results showed that the relative performance of SARIMA and Prophet depended on the specific validation period. Moreover, the aggregate April results slightly favored SARIMA, whereas Prophet achieved lower errors in several individual forecast windows. These variations indicate that model performance may depend on the temporal characteristics of the evaluation period, including unusual calendar-related patterns such as the Easter period. The Diebold–Mariano test, however, showed that these differences were not statistically significant. Therefore, the results do not support the conclusion that either model systematically outperforms the other in short-term ED attendance forecasting.

Overall, the findings demonstrate that ED attendance at the SCJU Craiova is characterized by systematic weekly variation, serial dependence, and calendar-related effects that can be incorporated into short-term forecasting models. SARIMA provides an explicit statistical representation of serial dependence and weekly seasonality, while Prophet offers an alternative additive forecasting framework incorporating weekly seasonality and holiday effects. Neither model demonstrated statistically superior predictive accuracy under the common out-of-sample validation framework employed in this study. Although SARIMA showed a marginal numerical advantage over the complete April 2026 period, this difference was not statistically significant. The findings therefore support the conclusion that SARIMA and Prophet provide comparable short-term predictive accuracy, while their different modeling structures may make one or the other more appropriate depending on the specific analytical or operational objective. This result also emphasizes the importance of combining conventional accuracy measures such as RMSE and MAPE with formal statistical comparisons when evaluating competing forecasting models for emergency healthcare demand.

## Figures and Tables

**Table 1:** Average daily ED visits across selected time intervals.

Time Interval	Average Visits	Observed Trend	Potential Contributing Factors
January (2025)	259.1	High level	Post-holidays, flu season and respiratory diseases
February–March	231.1	Lower level	Gradual decrease in viruses, normal activity.
April	248.2	Increase	Easter holidays, temperature variations.
May–June	260.7	High level	Craiova Days, IntenCity Festival, Bridge Days.
July–August	257.3	High level	Vacations, trauma related to recreational activities.
September–November	232.6	Lower level	School start, first waves of ARI/ILI.
December	235.7	Slight increase	Christmas, New Year’s Eve, bottlenecks in primary care.
January–March (2026)	234.4	Lower/stable level	A milder ARI/ILI season. Small temperature fluctuations.
April	251.5	Increase	Easter holidays, temperature variations.

**Table 2:** Daily descriptive statistics.

Day	Mean	Standard Deviation	Coef. of Variation	Skewness	Kurtosis	Minimum	Maximum
Monday	257.60	20.82	8.08%	−0.032	−0.251	205	299
Tuesday	236.72	24.76	10.46%	0.992	1.848	188	313
Wednesday	234.14	29.13	12.44%	0.207	−0.111	167	306
Thursday	233.50	28.84	12.35%	0.744	2.002	176	340
Friday	246.86	28.64	11.60%	0.549	0.199	186	327
Saturday	250.18	29.98	11.98%	0.704	0.191	202	330
Sunday	247.37	23.53	9.51%	−1.030	0.912	176	309

**Table 3:** Shapiro–Wilk test; authors’ contribution using EViews software 4.1, based on data from Table A5.

Day	Monday	Tuesday	Wednesday	Thursday	Friday	Saturday	Sunday
Shapiro–Wilk test (*p*-value)	0.176	0.000	0.904	0.011	0.216	0.012	0.712

**Table 4:** Pairwise Mann–Whitney U tests with unadjusted and Holm-adjusted *p*-values for comparisons of ED attendances across weekdays.

	Monday		Tuesday		Wednesday		Thursday		Friday		Saturday		Sunday	
	U Coef	*p*-Value	U Coef	*p*-Value pHolm	U Coef	*p*-Value pHolm	U Coef	*p*-Value pHolm	U Coef	*p*-Value pHolm	U Coef	*p*-Value pHolm	U Coef	*p*-Value pHolm
Mon	–	–	3712.0	<0.001 <0.001	3610.5	<0.001 <0.001	3688.0	<0.001 <0.001	3065.0	0.0036 0.0465	2883.5	0.0323 0.2264	3010.5	0.0073 0.0880
Tue			–	–	2497.5	0.7297 1.000	2521.0	0.6566 1.000	1833.0	0.0198 0.1585	1758.5	0.0081 0.0893	1604.0	0.0009 0.0171
Wed					–	–	2499.0	0.8413 1.000	1849.0	0.0172 0.1547	1708.5	0.0029 0.0408	1692.5	0.0023 0.0352
Thu							–	–	1779.0	0.0095 0.0951	1635.0	0.0010 0.0171	1634.0	0.0010 0.0171
Fri									–	–	2263.0	0.6183 1.000	2235.5	0.5369 1.000
Sat											–	–	2399.5	0.9372 1.000
Sun													–	–

**Table 5:** Augmented Dickey–Fuller test; authors’ contribution using EViews software, based on data from Table A5.

Null Hypothesis: SER01 Has a Unit Root			
		t-Statistic	Prob.
Augmented Dickey–Fuller test statistic		−4.605639	0.0001
Test critical values	1% level	−3.443805	
	5% level	−2.867367	
	10% level	−2.569936	

**Table 6:** SARIMA model with holiday dummy variable; authors’ contribution using EViews software, based on data from Table A5.

Dependent Variable: SER01				
Variable	Coefficient	Std. Error	t-Statistic	Prob.
C	242.8931	2.259888	107.4802	0.0000
HOLIDAY	18.57306	5.268297	3.525438	0.0005
AR(1)	0.299194	0.044091	6.785807	0.0000
MA(5)	0.141254	0.045256	3.121237	0.0019
SMA(7)	0.258638	0.044163	5.856397	0.0000
R-squared	0.254003	Akaike info criterion		9.217204
Adjusted R-squared	0.247773	Schwarz criterion		9.260407
Durbin-Watson stat.	1.991401	F-statistic		40.77342
		Prob(F-statistic)		0.000000
Breusch-Godfrey Serial Correlation LM Test:				
F-statistic	0.550934	Probability		0.576778
ARCH Test:				
F-statistic	0.212781	Probability		0.644805

**Table 7:** RMSE and MAPE for SARIMA with a holiday dummy variable.

Model	Origin 1	Origin 2	Origin 3	Origin 4
	1–7 April 2026	8–15 April 2026	16–22 April 2026	23–30 April 2026
SARIMA RMSE	16.93	30.16	18.96	20.93
SARIMA MAPE	4.70%	11.03%	6.54%	7.13%

**Table 8:** RMSE and MAPE for Prophet without yearly seasonality.

Model	Origin 1	Origin 2	Origin 3	Origin 4
	1–7 April 2026	8–15 April 2026	16–22 April 2026	23–30 April 2026
Prophet RMSE	15.61	35.30	15.00	20.65
Prophet MAPE	5.48%	13.22%	5.13%	5.89%

**Table 9:** Comparative RMSE and MAPE across the four rolling-origin validation windows.

Model	Origin 1	Origin 2	Origin 3	Origin 4
	1–7 April 2026	8–15 April 2026	16–22 April 2026	23–30 April 2026
SARIMA RMSE	16.93	30.16	18.96	20.93
Prophet RMSE	15.61	35.30	15.00	20.65
SARIMA MAPE	4.70%	11.03%	6.54%	7.13%
Prophet MAPE	5.48%	13.22%	5.13%	5.89%

**Table 10:** Aggregate forecasting accuracy over 1–30 April 2026.

Model	RMSE	MAPE
SARIMA	22.59	7.46%
Prophet	23.54	7.56%

**Table 11:** Diebold–Mariano test with HAC/Newey–West correction.

Statistic	Result
Evaluation period	1–30 April 2026
Loss function	Squared forecast error
Mean loss differential SARIMA−Prophet	−44.21
Newey–West lag	1
HAC standard error	77.92
DM statistic	−0.567
*p*-value	0.575

## Data Availability

The original contributions presented in this study are included in the article. Further enquiries can be directed to the corresponding author.

## Appendix A

**Table A1:** Correlogram; authors’ contribution using EViews software, based on data from Table A5.

Included Observations: 485						
Autocorrelation	Partial Correlation		AC	PAC	Q-Stat	Prob
.|***|	.|***|	1	0.424	0.424	87.914	0.000
.|**|	.|.|	2	0.225	0.055	112.75	0.000
.|* |	.|*|	3	0.187	0.090	129.90	0.000
.|**|	.|*|	4	0.212	0.117	151.87	0.000
.|**|	.|*|	5	0.235	0.114	179.09	0.000
.|**|	.|*|	6	0.273	0.140	215.90	0.000
.|***|	.|**|	7	0.358	0.215	279.31	0.000
.|**|	.|.|	8	0.263	0.019	313.59	0.000
.|*|	.|.|	9	0.151	−0.039	324.91	0.000
.|*|	.|.|	10	0.125	−0.008	332.71	0.000
.|*|	.|.|	11	0.149	0.012	343.82	0.000
.|*|	.|.|	12	0.166	0.009	357.51	0.000
.|*|	.|.|	13	0.199	0.048	377.36	0.000
.|**|	.|*|	14	0.237	0.068	405.55	0.000
.|*|	.|.|	15	0.178	−0.006	421.41	0.000
.|*|	.|.|	16	0.095	−0.035	426.00	0.000
.|.|	.|.|	17	0.045	−0.056	427.03	0.000
.|*|	.|.|	18	0.070	−0.014	429.53	0.000
.|*|	.|*|	19	0.169	0.092	444.01	0.000
.|*|	.|.|	20	0.168	0.011	458.31	0.000
.|**|	.|*|	21	0.213	0.094	481.46	0.000
.|*|	.|.|	22	0.144	−0.010	491.98	0.000
.|*|	.|.|	23	0.116	0.039	498.83	0.000
.|.|	*|.|	24	0.010	−0.102	498.89	0.000
.|*|	.|.|	25	0.049	0.007	500.14	0.000
.|*|	.|.|	26	0.121	0.013	507.69	0.000
.|*|	.|.|	27	0.117	−0.023	514.79	0.000
.|*|	.|.|	28	0.116	0.000	521.77	0.000
.|.|	*|.|	29	0.030	−0.081	522.25	0.000
.|.|	.|.|	30	0.022	−0.006	522.50	0.000
.|.|	.|.|	31	0.029	0.031	522.93	0.000
.|.|	.|.|	32	0.000	−0.057	522.93	0.000
.|.|	.|.|	33	0.049	0.004	524.17	0.000
.|*|	.|.|	34	0.079	0.017	527.46	0.000
.|*|	.|.|	35	0.103	0.053	533.05	0.000
.|*|	.|*|	36	0.094	0.071	537.68	0.000

*, **, and *** indicate the level of statistical significance of autocorrelations (*—significant at ≈10%; **—significant at ≈5%; ***—significant at ≈1%).

**Table A2:** Forecast using the SARIMA model with a holiday dummy variable; authors’ contribution using EViews software, based on data from Table A5.

Data	ED Patients	Forecast	Lower Limit	Upper Limit
1 April 2026	221	227.35	180.73	273.97
2 April 2026	253	244.70	196.73	292.67
3 April 2026	269	231.94	183.87	280.00
4 April 2026	259	239.66	191.57	287.75
5 April 2026	241	242.24	194.06	290.43
6 April 2026	248	248.43	199.80	297.06
7 April 2026	253	240.90	192.19	289.62
8 April 2026	263	246.30	199.90	292.70
9 April 2026	242	246.75	198.95	294.54
10 April 2026	236	270.38	222.49	318.26
11 April 2026	211	248.03	200.12	295.93
12 April 2026	228	261.04	213.02	309.06
13 April 2026	297	260.46	211.97	308.94
14 April 2026	286	244.82	196.25	293.39
15 April 2026	261	243.70	193.42	293.98
16 April 2026	241	239.04	192.49	285.58
17 April 2026	252	232.08	183.99	280.17
18 April 2026	260	239.07	190.88	287.25
19 April 2026	269	241.49	193.30	289.68
20 April 2026	261	255.26	206.97	303.54
21 April 2026	226	253.60	204.84	302.35
22 April 2026	257	245.72	196.88	294.57
23 April 2026	259	248.73	202.31	295.15
24 April 2026	228	250.81	202.80	298.81
25 April 2026	264	248.13	200.02	296.23
26 April 2026	289	247.30	199.19	295.42
27 April 2026	267	244.92	196.70	293.15
28 April 2026	243	236.05	187.35	284.75
29 April 2026	231	244.87	196.09	293.64
30 April 2026	231	243.62	193.35	293.88
1 May 2026		254.81	208.51	301.12
2 May 2026		248.43	200.51	296.36
3 May 2026		251.73	203.72	299.75
4 May 2026		245.06	197.03	293.08
5 May 2026		240.95	192.82	289.08
6 May 2026		237.60	189.03	286.16
7 May 2026		237.96	189.33	286.60

**Table A3:** Forecast using the Prophet model without yearly seasonality; authors’ contribution using Prophet (Python software 3.13.15), based on data from Table A5.

Data	ED Patients	Forecast	Lower Limit	Upper Limit
1 April 2026	221	231.84	180.36	281.62
2 April 2026	253	229.97	180.39	280.82
3 April 2026	269	245.09	194.19	295.81
4 April 2026	259	250.10	198.42	301.59
5 April 2026	241	245.32	194.50	295.58
6 April 2026	248	255.85	204.29	308.47
7 April 2026	253	235.17	185.21	284.97
8 April 2026	263	231.68	180.33	281.47
9 April 2026	242	230.34	180.45	279.47
10 April 2026	236	276.43	226.06	328.66
11 April 2026	211	250.24	202.76	298.86
12 April 2026	228	273.75	222.46	324.53
13 April 2026	297	284.23	233.42	336.26
14 April 2026	286	235.44	184.85	286.70
15 April 2026	261	231.68	180.05	282.44
16 April 2026	241	230.53	180.93	279.40
17 April 2026	252	245.48	193.86	296.57
18 April 2026	260	249.65	199.14	300.88
19 April 2026	269	244.80	195.18	295.02
20 April 2026	261	256.18	205.75	306.71
21 April 2026	226	236.21	184.74	285.80
22 April 2026	257	232.59	180.94	283.15
23 April 2026	259	230.69	180.23	281.45
24 April 2026	228	245.59	196.35	294.55
25 April 2026	264	249.81	198.77	299.56
26 April 2026	289	245.17	194.77	294.31
27 April 2026	267	256.26	205.16	306.84
28 April 2026	243	236.06	186.59	288.10
29 April 2026	231	232.95	183.72	281.92
30 April 2026	231	230.69	180.81	281.27
1 May 2026		287.12	236.42	337.91
2 May 2026		250.01	199.11	301.72
3 May 2026		245.85	195.52	295.59
4 May 2026		256.44	206.28	307.28
5 May 2026		236.17	183.94	285.17
6 May 2026		232.92	182.80	283.94
7 May 2026		231.11	180.62	283.06

**Table A4:** Robustness of the Diebold–Mariano test to the HAC lag specification.

Newey–West Lag	DM Statistic	*p*-Value
1	−0.567	0.575
2	−0.589	0.560
3	−0.597	0.555
4	−0.587	0.562
5	−0.614	0.544
6	−0.636	0.530
7	−0.667	0.510

**Table A5:** Daily ED visits: January 2025–April 2026.

Data	1	2	3	4	5	6	7	8	9	10	11	12	13	14	15	16	17	18	19	20	21	22	23	24	25	26	27	28	29	30	31
January 2025	265	340	314	319	309	281	271	294	263	227	229	251	274	244	215	196	261	268	220	235	232	245	255	292	270	252	258	232	259	259	205
February 2025	242	239	251	214	180	222	217	204	220	248	227	205	220	238	212	233	238	241	216	194	202	256	221	233	230	197	217	231			
March 2025	229	222	233	233	239	236	220	220	260	265	234	236	221	224	264	240	268	188	236	250	243	262	232	247	241	241	250	237	231	245	241
April 2025	231	209	213	243	272	212	265	244	192	217	235	261	257	254	241	222	219	287	224	259	299	313	277	249	281	223	232	273	246	298	
May 2025	273	300	265	269	284	238	251	261	259	260	239	267	228	253	241	219	226	244	251	224	260	242	276	222	248	277	236	274	249	242	259
June 2025	273	242	284	236	221	248	308	279	299	297	286	249	272	279	295	249	282	252	247	296	281	244	292	232	236	251	274	330	285	250	
July 2025	251	306	313	252	251	276	267	242	222	252	253	242	249	285	233	250	218	241	244	242	287	231	237	273	305	252	257	285	250	234	218
August 2025	243	281	257	265	312	248	260	238	276	265	270	266	284	238	241	298	262	283	237	240	271	249	252	266	252	227	206	206	261	288	298
September 2025	246	263	233	269	259	256	245	245	249	237	247	283	242	267	275	246	184	235	225	235	266	241	218	242	222	253	238	195	205	205	
October 2025	216	185	186	218	238	206	199	213	243	201	251	256	257	198	227	230	227	222	260	258	228	229	246	245	228	239	283	212	195	247	221
November 2025	254	239	282	199	246	214	224	219	236	247	217	237	192	216	235	227	240	247	219	213	231	221	220	255	234	202	176	237	252	247	
December 2025	247	255	221	213	232	202	254	238	220	218	208	248	218	241	259	212	195	217	256	264	255	249	217	194	208	288	326	279	277	230	167
January 2026	235	327	312	251	282	237	249	210	267	258	239	241	191	219	248	234	236	216	227	242	217	230	229	227	236	241	231	251	194	212	230
February 2026	176	253	227	207	231	249	247	225	244	217	221	196	204	202	233	248	231	220	184	247	216	208	233	227	256	211	236	220			
March 2026	242	227	224	196	193	233	292	232	237	227	270	231	273	278	256	239	210	258	222	272	247	264	275	240	214	263	208	223	248	277	241
April 2026	221	253	269	259	241	248	253	263	242	236	211	228	297	286	261	241	252	260	269	261	226	257	259	228	264	289	267	243	231	231	
